# Skin as outermost immune organ of vertebrates that elicits robust early immune responses after immunization with glycoprotein of spring viraemia of carp virus

**DOI:** 10.1371/journal.ppat.1012744

**Published:** 2024-12-09

**Authors:** Zhao Zhao, Liang Zhao, Xue-Feng Wei, Yi-Jun Jia, Bin Zhu

**Affiliations:** 1 College of Animal Science and Technology, Northwest A&F University, Yangling, Shaanxi, China; 2 Engineering Research Center of the Innovation and Development of Green Fishery Drugs, Universities of Shaanxi Province, Northwest A&F University, Yangling, Shaanxi, China; Thomas Jefferson University, UNITED STATES OF AMERICA

## Abstract

As the outermost immune organ in vertebrates, the skin serves as the primary interface with the external environment and plays a crucial role in initiating the early immune response. The skin contains a variety of immune cells that induce mucosal and systemic immune responses, rendering it a prime target for vaccination strategies. Insight into the mechanisms through which vaccination triggers early immune responses is paramount for advancing animal and human health, yet our current understanding remains limited. Given its significance in vertebrate evolution, teleost fish emerges as an excellent model for investigating the early immune response of skin. In this study, we demonstrate that significant quantities of vaccine can be absorbed by the skin and transported to the body through dermis and muscle metabolism by immerses immune zebrafish with glycoprotein of spring viraemia of carp virus. Immersion immunization can elicit robust and enduring immune protection, with the skin triggering a potent immune response early in the immunization process. Analysis of the skin transcriptome revealed the involvement of numerous immune-related genes in the immersion immune response, with indications that HSP70 and MAPK signals might play pivotal roles in the immune process induced by glycoprotein. Co-immunoprecipitation and cell co-localization studies confirmed the interaction between glycoprotein and HSP70. Subsequent research demonstrated that overexpression or inhibition of HSP70 could respectively enhance or impede the expression of JNK and related proteins. However, the survival rate and immune response of HSP70 inhibited zebrafish with glycoprotein treatment were significantly reduced. These findings propose that the interaction between glycoprotein and HSP70 may activate JNK, thereby modulating mucosal and systemic immune responses induced by glycoprotein. This investigation offers novel insights and a foundational understanding of early skin immune reactions.

## Author summary

The skin is usually the first barrier through which vertebrates come into contact with the external environment, playing a very important role in protecting the body against external pathogen infections. In recent years, researchers have noticed that immune responses through the skin can effectively enhance the ability to resist pathogens, but how it regulates the immune mechanisms is still unclear. As a classic model animal among vertebrates, zebrafish plays an important role in studying skin immune responses and mechanisms due to its unique living environment and high similarity to the human immune system. This study found that zebrafish skin can absorb a large amount of glycoprotein vaccine after immersion immunization, and quickly induce a strong adaptive immune response in the skin shortly after immunization. Subsequent research demonstrated that glycoprotein interacts with HSP70 to regulate MAPK through JNK, thereby modulating mucosal and systemic immune responses. In addition, HSP70-inhibited zebrafish showed a significant reduction in survival rate after viral challenge and immune responses. Our research provides important insight for revealing the early skin immune mechanisms of vertebrates.

## Introduction

The skin of vertebrates serves as a protective barrier against a range of environmental factors and plays a crucial role in systemic and mucosal responses [1]. The immune system of the skin is mainly composed of the epidermis and dermis and contains several skin resident immune cells, such as Langerhans cells and keratinocytes which are mainly distributed in the epidermis, various subsets of dendritic cells (DCs), mast Cells, macrophages, and several T cell types, which are mainly distributed in the dermis [2]. The upregulation of MHC II on keratinocytes and Langerhans cells facilitates the infiltration of white blood cells into the skin. Mast cells, capable of expressing both MHC I and MHC II, enhance antigen presentation by expressing co-stimulatory molecules like CD80 and CD86, thereby influencing T cell aggregation. Moreover, mast cells serve as antigen-presenting cells, activating antigen-specific T cells and B cells, and thereby eliciting mucosal and systemic immune responses [3–5]. Due to present antigens through the skin is very easy to participate in the skin immune system, immunization through the skin route is an attractive route for vaccine delivery [6]. Transdermal administration prompts a strong mucosal immune response, DeMuth et al demonstrated that delivering an HIV vaccine to the skin of mice induces mucosal and systemic immune responses comparable to intramuscular injection [7]. The inactivated porcine reproductive and respiratory syndrome virus (PRRSV) vaccine was inoculated on the skin of pigs with dissolved microneedle patches, the results showed that systemic inflammation and immune cell response were induced by cutaneous immunization, especially the early immune response was observed in the skin [8].

Teleosts have played a pivotal role in the evolution of vertebrates, particularly due to their immune system being the most primitive among vertebrates. As a result, teleosts are considered the ideal model for studying the immune evolution and regulatory mechanisms in vertebrates [9]. Unlike mammalian skin, which primarily serves as a protective barrier, teleost fish have evolved mucous surfaces that secrete mucus, including lymphatic tissue associated with the skin, to adapt to their aquatic environment [10]. The skin of fish, functioning as a mucosal surface in direct contact with water, contains significant populations of immune cells such as B cells, T cells, macrophages, and granulocytes, highlighting its role in immune defense through mucosal secretions [11,12]. Immersion vaccination in fish elicits immune responses by stimulating the mucosal tissues of the skin, which have been shown to exhibit significant immune activity [13]. A study of *Flavobacterium columnare* demonstrated that immersion inoculation with a bionic adhesive nanovaccine induces a robust mucosal immune response, IgM antibody titer was significantly higher than that of the control group at 14 days post-vaccination, and IgM and IgT genes in the spleen of immunized fish showed significant upregulation [14]. Klafack et al sed an alginate encapsulated attenuated live virus (KHV) as a vaccine and observed enhanced specific antibody responses in common carp through immersion vaccination, resulting in robust and reliable immunity [15]. Nevertheless, the mechanism underlying the initiation of early immune responses through the skin during immersion vaccination remains unclear.

The skin has been established as the optimal tissue for delivering vaccine antigens into the draining vein, thereby eliciting immune effects. Direct injection of antigens into the draining lymph nodes induces antibody responses comparable to those of the intradermal vaccine, and lymph node resident immune cells such as B cells, dendritic cells, and macrophages interact with the antigens [16]. In a study immunizing mice with an influenza virus vaccine via both a skin microneedle patch and intramuscular injection, mice in the skin microneedle patch group detected the higher IgG1 antibody response and enhanced cellular immune response, including the increased number of IL-4 and IFN-γ producing cells and higher frequency of antigen-specific plasma cells compared to the intramuscular injection group [17]. Teleost fish have long been regarded as valuable model organisms for investigating skin immune mechanisms over the course of evolutionary history, shedding light on the development of mucosal immune systems [18]. Immunoglobulin Z (IgZ) is extensively present in the skin and other mucosal lymphatic tissues of fish, with its production primarily reliant on αβ T/CD4^+^ cells, moreover, IgZ^+^ B cells are widely dispersed throughout the primary and secondary lymphoid tissues of the entire organism [19]. The iTRAQ and LC-MS/MS were used to analyze skin differential proteomics after *Vibrio vulnificus* infection with *Cynoglossus semilaevis*, and several immune-related differentially expressed proteins such as HSP70, C3, C5, C9 and CD59 were screened. In addition, KEGG enrichment analysis revealed that most of the identified immune proteins were significantly associated with complement and coagulation cascades, antigen processing and presentation, salivary secretion, and phagosome pathways [20]. Limited understanding exists regarding the molecular mechanisms of early skin immunity, particularly concerning how vaccines elicit mucosal and adaptive immune responses via the skin.

Heat shock proteins (HSPs) are known to be phylogenetically conserved across organisms from bacteria to humans, functioning as chaperone molecules to aid in protein stabilization, facilitate protein folding, and uphold or restore proper conformation [21,22]. Among them, HSP70 is a well-studied heat shock protein, which can play an important role in cellular stress response, signal transduction regulation and antiviral immunity through interaction with other proteins [23,24]. The recombinant *Megalobrama amblycephala* Hsp70 protein (rMaHsp70) has demonstrated the ability to enhance proliferation, suppress apoptosis in *M*. *amblycephala* fin cells (MAF), and induce widespread expression of HSP70 throughout the organism, thereby eliciting a robust immune response via HSP70 protein immunization [25]. In addition, HSP70 has also been shown to interact with viral or host proteins to regulate pathway signaling responses and thus participate in immune responses. HSP70 is prominently expressed in numerous types of tumors and it has the capability to counter cisplatin-induced apoptosis in HGC-27 cells by modulating the mitogen-activated protein kinase (MAPK) signaling pathway. Importantly, the upregulation of HSP70, achieved via plasmid transfection to overexpress HSP70, can mitigate HGC-27 cell apoptosis instigated by cisplatin [26]. Immunoprecipitation assay demonstrated that grouper heat shock cognate protein 70 (GHSC70) interacts with Nervous necrosis virus (NNV) capsid protein, and knocking down the expression of GHSC70 gene with siRNA in NNV-infected GF-1 cells can significantly down-regulate the expression of viral RNA. The adsorption capacity of GHSC70 antiserum pretreated cells to NNV was significantly reduced, suggesting that GHSC70 may be involved in NNV entry of GF-1 cells as an NNV receptor or co-receptor protein [27]. While further research confirms the close relationship between HSP70 and the host immune system, the precise mechanisms are not yet fully elucidated.

Given its significance in vertebrate evolution, teleost fish emerges as an excellent model for investigating the early immune response of skin. Spring viraemia of carp virus (SVCV) is a type of *Rhabdoviridae* that is widely prevalent worldwide and can infect the vast majority of cyprinid fish [28,29]. SVCV entering the host, primarily through mucosal surfaces such as the skin, gills, and digestive tract, leads to rapid mortality in infected fish [30,31]. The skin, as the initial entry point of the virus, may play a crucial role in exploring its early immune response. In this work, we immunized zebrafish with glycoprotein from the SVCV as animal models to investigate the underlying mechanisms of skin early immune response. Our study indicated that HSP70 plays an important role in the regulation of adaptive mucosal and systemic immune responses in teleost fish, and offers valuable insights to understand the skin immune mechanisms of vertebrates.

## Results

### Skin induces strong early immunoprotection against SVCV by uptake G3 protein after immersion vaccination

**Fig 1:**
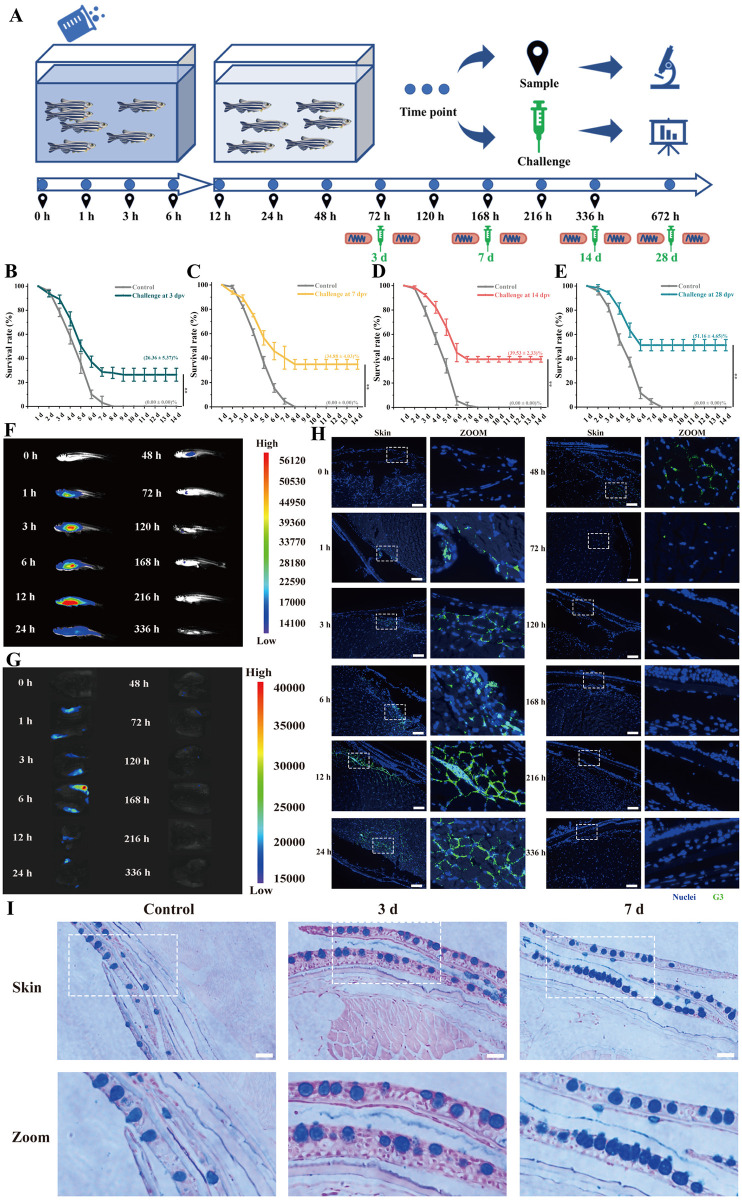
Strong immunoprotection in skin elicited by G3 against SVCV after immersion vaccination. (A) Schematic diagram of *in vivo* uptake and metabolism analysis, sampling, and challenge of zebrafish after immersion vaccination by G3. Zebrafish were immersion vaccinated with G3 vaccine for 6 h. Subsequently, the vaccinated fish were transport to clean tanks. (B) After 3 days post-vaccination, the survival rates of immunized fish were tested for virus challenge. (C) After 7 days post-vaccination, the survival rates of immunized fish were tested for virus challenge. (D) After 14 days post-vaccination, the survival rates of immunized fish were tested for virus challenge. (E) After 28 days post-vaccination, the survival rates of immunized fish were tested for virus challenge. (F) Representative *in vivo* fluorescence images of vaccinated fish at different time points. The fluorescence intensity represents the amount of G3 vaccine detected. (G) Representative *in vivo* fluorescence images of skin in vaccinated fish at different time points. The fluorescence intensity represents the amount of G3 vaccine detected. (H) Representative *in vivo* fluorescence images of frozen section in skin at different time points. G3 vaccine were stained with FITC (green), nuclei were stained with DAPI (blue). Scale bars, 50 μm. (I) Histological examination by AB staining of skin from vaccinated fish at 3 dpv and 7 dpv. Scale bars, 50 μm. Fig 1A used some open-source artworks from Open Clipart and Wikimedia Commons. Science Beaker–Green (https://openclipart.org/detail/307196/science-beaker-green) and arrow next (https://openclipart.org/detail/12603/arrow-next) were used from Open Clipart under Creative Commons Zero 1.0 license (https://creativecommons.org/publicdomain/zero/1.0/), and no change were made to original artwork. Yellow test tube icon (https://commons.wikimedia.org/wiki/File:Yellow_test_tube_icon.svg), Microscope icon (black and blue) (https://commons.wikimedia.org/wiki/File:Microscope_icon_(black_and_blue).svg), and Analysis—The Noun Project (https://commons.wikimedia.org/wiki/File:Analysis_-_The_Noun_Project.svg) were used from Wikimedia Commons under Creative Commons CC0 1.0 Universal Public Domain Dedication license (https://creativecommons.org/publicdomain/zero/1.0/), and no change were made to original artwork. Syringe—Lorc—game-icons (https://commons.wikimedia.org/wiki/File:Syringe_-_Lorc_-_game-icons.svg) were was used from Wikimedia Commons under Creative Commons Attribution 3.0 Unported license (https://creativecommons.org/licenses/by/3.0/), and no change were made to original artwork. 201108 zebrafish (https://commons.wikimedia.org/wiki/File:201108_zebrafish.png) was used from Wikimedia Commons under Creative Commons Attribution 4.0 International license (https://creativecommons.org/licenses/by/4.0/), and no change were made to original artwork. Except for these open-source artworks, other parts in Fig 1A were drawn by ourselves.

In previous studies in our laboratory, G3 (glycoprotein, G^251-381^) protein has a higher antibody titer than the other three proteins, has been demonstrate that may be a potential epitope that plays an important role in SVCV, and may be use it as antigen to construct a highly effective vaccine [32]. Following bath immunization of zebrafish with the purified recombination His-G3 protein (S1A Fig) for 6 hours, they were transferred to clean water for subsequent feeding. After 6 hours of immersion vaccination, challenge tests were conducted on zebrafish at different times post vaccination to evaluate the immunoprotection efficacy induced by G3 protein (Fig 1A). The challenge result showed the mortality rates of 100% in control group, The results of the challenge revealed a 100% mortality rate in the control group, while the G3 group exhibited improved survival rates, with 26.36 ± 5.37% at 3 days post-vaccination (dpv), 34.88 ± 4.03% at 7 dpv and 39.53 ± 2.33% compared to the control group. Notably, the highest survival rate (51.16 ± 4.65%) was observed at 28 dpv (Fig 1B–1E). Moreover, survival analyses used log-rank test indicated that there were significant differences between the immune group and the control group, even at 3 dpv (S2 Fig). Viral load tests by qPCR showed similar results (S3A and S3B Fig), the significantly lower viral loads of G3 group in the spleen (~0.8 fold) compared to the control group at 7 dpv, along with notably reduced virus titers of G3 group in the skin (~0.8 fold) compared to the control group at 3 dpv. Analyses of acid phosphatase assay (ACP), superoxide dismutase activity (SOD), alkaline phosphatase assay (AKP), and complement C3 activity in G3 groups indicated significantly higher levels than those in the control groups at 3 and 7 dpv (S3C–S3F Fig). These findings indicate that G3 protein can induce a strong immune response at 3 and 7 dpv by immersion vaccination, enhancing the ability against pathogens.

The metabolism of G3 immersion immunity in the organism was investigated using macrophages and zebrafish as subjects, respectively. For the *in vitro* evaluation, macrophages were incubated with G3 for varying durations and subsequently examined using a fluorescence microscope, assessment of cell viability confirmed that the uptake of G3 did not exert toxic effects on the macrophages after co culturing macrophages with G3 for 12 hours (S1B Fig). The macrophages could internalize G3 after co-incubation for 3 hours, with increased uptake observed at 6 hours and 12 hours of incubation (S1C Fig). For the *in vivo* evaluation, the fluorescence intensity exhibited a gradual increase in fish from 0 hours to 6 hours after immunization, followed by a significant decrease at 48 hours, with only minimal fluorescence observed at 72 hours (Fig 1F). In addition, strong fluorescence was detected on the skin of the zebrafish within the initial 24 hours following immunization. Subsequently, skin samples were separately obtained for *in vivo* imaging analysis, revealing a progressive increase in G3 uptake in the skin post-immunization. The fluorescence intensity exhibited a gradual decline after 12 hours and became nearly undetectable by 48 hours (Fig 1G). The skin of zebrafish was observed by fluorescence microscope at different time points, and bright green fluorescence could be observed in the epidermis and dermis of the skin at 3 h and 6 h after inoculation (Fig 1H). Subsequently, substantial green fluorescence was noted in the dermis and muscles from 12 hours up to 48 hours after inoculation. Furthermore, after 14 days of immunization, no significant lesions or abnormalities were observed in the HE-stained sections of the spleen, kidney, and skin. (S3G Fig). In combination with AB (Alcian Blue) staining, a notable increase in the number of mucus cells in skin were conspicuous increase at 3 and 7 dpv (Fig 1I). These findings suggest a significant uptake of G3 into the skin post-immunization, and induce a certain immune response on the skin.

### Adaptive immune response in skin after immersion immunization

**Fig 2:**
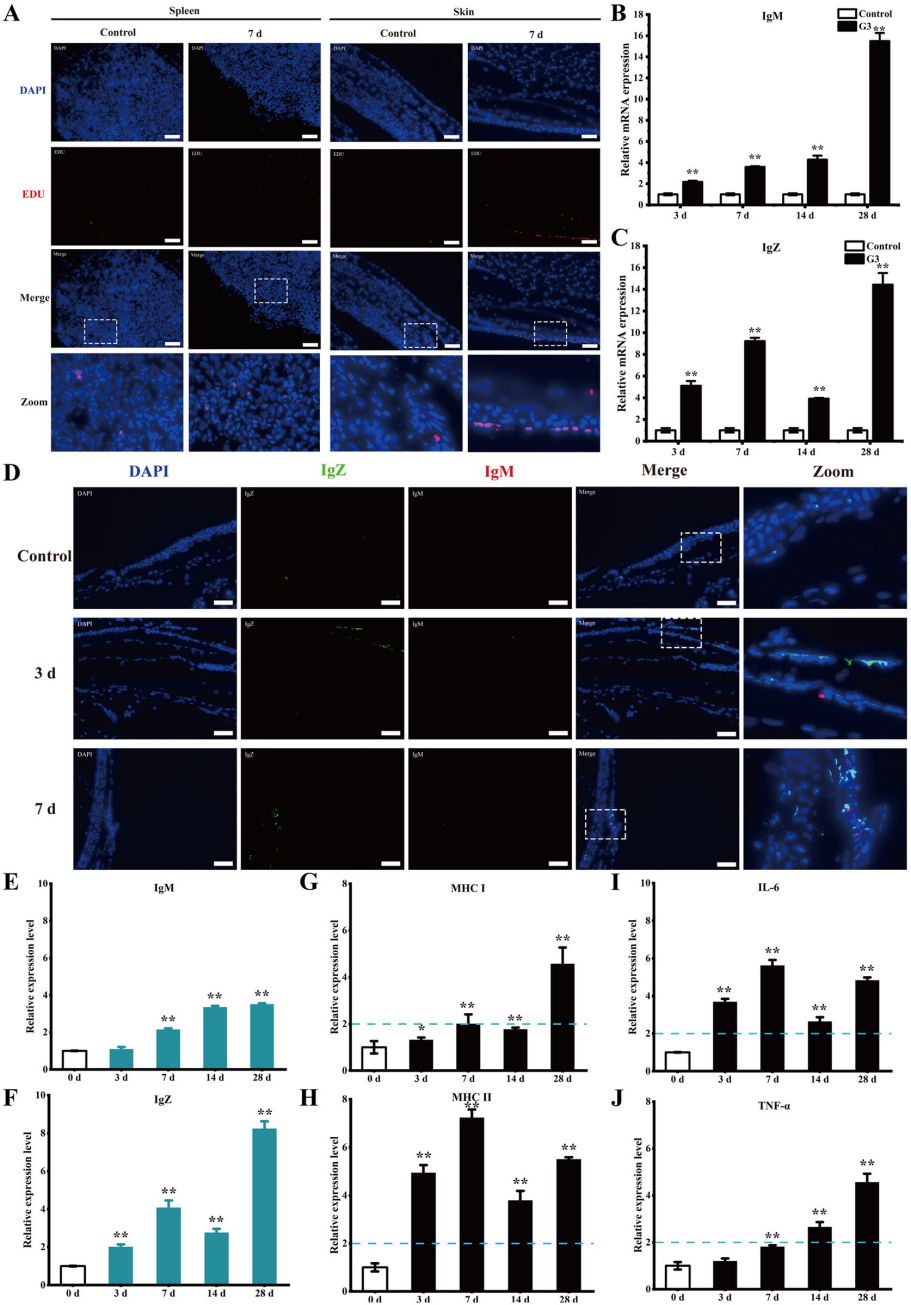
Adaptive immune response in skin after immersion immunization. (A) Immunofluorescence analysis was performed to assess cell proliferation through EdU incorporation in the skin of vaccinated fish. EdU (red) was used to detect the cell proliferation in the skin tissue sections, nuclei were stained with DAPI (blue). Scale bars, 50 μm. (B) Relative mRNA levels of *IgM* in skin from vaccinated fish at different time points were examined by qPCR. (C) Relative mRNA levels of *IgZ* in skin from vaccinated fish at different time points were examined by qPCR. (D) Representative immunofluorescence images for IgZ (green) and IgM (red) of skin from vaccinated fish on days 3 and 7 after immunization. Nuclei were stained with DAPI (blue). Scale bars, 50 μm. (E) ELISA examined the relative protein levels of IgM in skin from vaccinated fish at different time points. (F) ELISA examined the relative protein levels of IgZ in skin from vaccinated fish at different time points. (G) ELISA examined the relative protein expression levels of MHC I in skin from vaccinated fish at different time points. The expression levels of MHC I at 3 dpv (1.31 ± 0.1) and 7 dpv (2.0 ± 0.41) were significantly higher than control group. (H) ELISA examined the relative protein expression levels of MHC II in skin from vaccinated fish at different time points. The expression levels of MHC II at 3 dpv (4.94 ± 0.32) and 7 dpv (7.23 ± 0.35) were significantly higher than control group. (I) ELISA examined the relative protein expression levels of IL-6 in skin from vaccinated fish at different time points. The expression levels of IL-6 at 3 dpv (3.69 ± 0.16) and 7 dpv (5.62 ± 0.3) were significantly higher than control group. (J) ELISA examined the relative protein expression levels of TNF-α in skin from vaccinated fish at different time points. The expression levels of TNF-α at 7 dpv (1.81 ± 0.06) were significantly higher than control group. The P-value for each study was calculated using one-way ANOVA. Statistical significance is indicated as follows: *P < 0.05, **P < 0.01. The data presented are representative of three independent experiments, with means ± standard error of the mean (SEM).

To further analyze the immune response of skin after G3 immunization, we investigated B cell responses, Igs, and APCs maturation in skin of zebrafish. Enhanced cell proliferation was observed in both spleen and skin at 7 dpv compared to the control group, with significantly greater proliferation detected in the skin (Figs 2A and S4A). Using qPCR analysis, the expression of IgM in skin was found to be significantly upregulated with about 2-fold at 3 dpv (Fig 2B), and the expression of IgZ was upregulated with about 5-fold (Fig 2C). Using immunofluorescence analysis, minimal IgM^+^ B cells were found in the skin of control fish, and no IgZ^+^ B cells was observed (Figs 2D, S4B and S4C). At 3 and 7 dpv, the presence of IgM^+^ B cells is still rare, but the presence of IgT^+^ B cells showed obvious increase when compared with control fish. To evaluate the skin Ig responses, ELISA analysis was conducted to determine the relative protein expression of IgM and IgZ (Fig 2E and 2F). A moderate (∼2-fold) yet significant increase in IgZ was observed at 3 dpv, with about a 4-fold increase noted at 7 dpv. Conversely, a substantial (∼2-fold) increase in IgM was only observed at 7 days, with no increase detected at 3 dpv. Subsequently, the maturation of APCs and cytokine levels of skin were assessed through ELISA analysis (Fig 2G–2J), the expression of mature APCs markers (MHC-I and MHC-II) and cytokines (TNF-α and IL-6) showed a significant increase at 3 and 7 dpv. These findings indicate that the skin can elicit a robust adaptive immune response even as early as 3 days post-immunization.

### Transcriptome analysis provides insights into the immune response in skin

**Fig 3:**
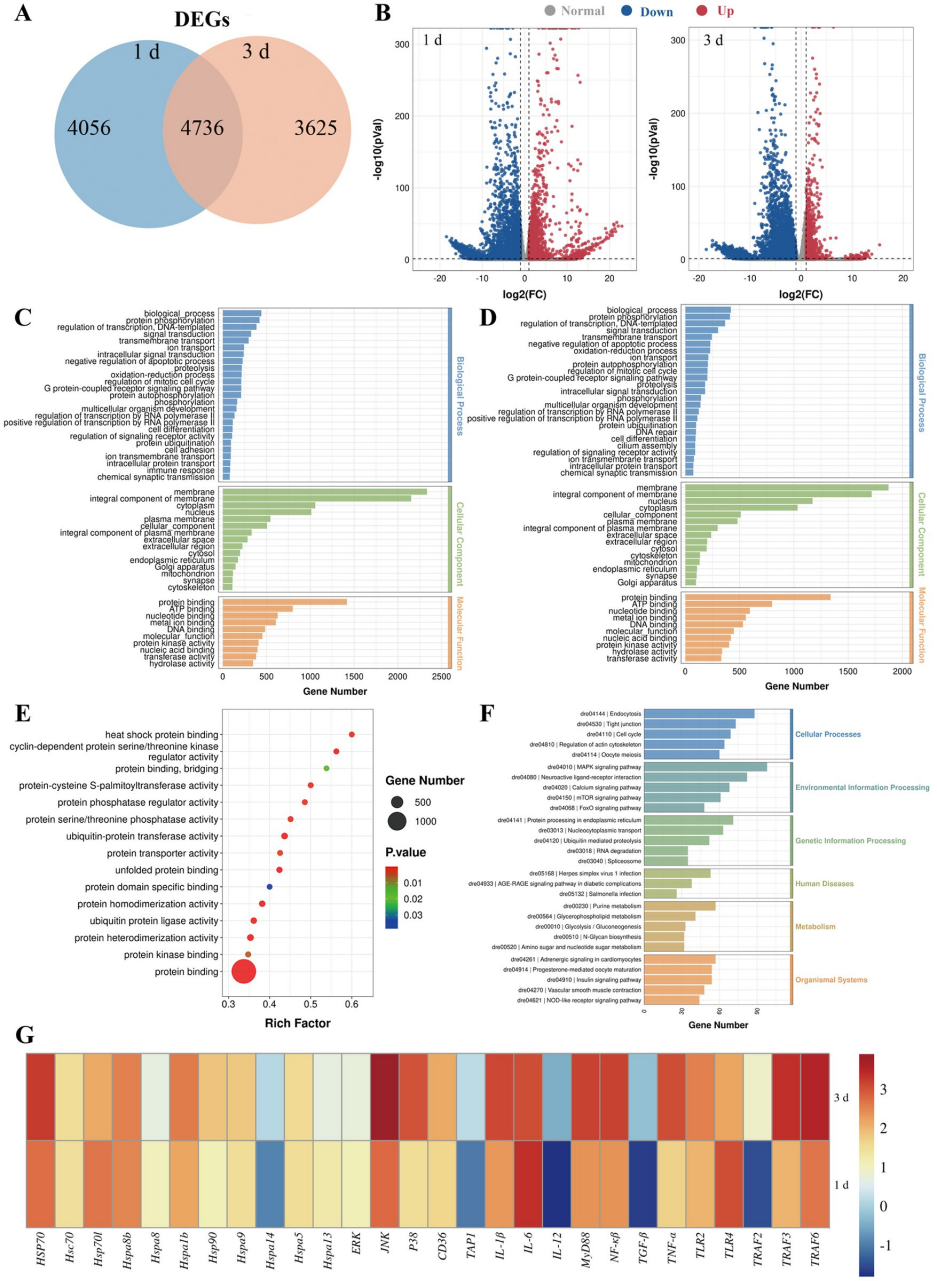
Transcriptome analysis provides insights into the immune response in skin. (A) Venn diagram showing the number of the statistical results of DEGs in the skin of zebrafish at 1 and 3 dpv. (B) Volcano plot displaying the DEGs distribution of significantly upregulated (expression fold change >2 and FDR <0.05, red spots) and downregulated (expression fold change <2 and FDR <0.05, blue spots) at 1 and 3 dpv compared with control group. (C) The GO enrichment for differentially expressed genes at 1 dpv compared with control group. (D) The GO enrichment for differentially expressed genes at 3 dpv compared with control group. (E) Molecular function that were significantly altered in skin of vaccinated fish at 1 dpv compared to 3 dpv were revealed through RNA-seq studies. (F) The DEGs enrichment KEGG pathway analysis at 1 dpv compared with 3 dpv. (G) qPCR analysis on the expression of related with heat shock protein binding, MAPK signaling pathway, and immune response gene in skin from vaccinated fish at 1 and 3 dpv.

G3 effectively induces robust mucosal immunity at the early stage of vaccination, particularly at 3 dpv. Therefore, we selected 1 dpv and 3 dpv for subsequent high-throughput transcriptome sequencing analysis. RNA-Seq libraries were constructed from nine samples that separately represented three groups (Control, 1 d, and 3 d groups contain three samples each) which were sequenced on an Illumina platform. After G3 immersion vaccination, we found significant modifications in mRNA expression, with 8792 differentially expressed genes (DEGs) at 1 dpv and 8361 DEGs at 3 dpv, respectively (Fig 3A and 3B). To validate the DEGs identified by RNA-seq, we randomly chose 5 upregulated and 5 downregulated genes for qRT-PCR confirmation. The results demonstrated a significant correlation between the expression values determined by RNA-seq and qPCR at each time point (S5A–S5D Fig), indicating that the RNA-seq results were as accurate as those obtained by qPCR in determining gene expression *in vivo*.

Gene ontology (GO) functional enrichment analysis for DEGs at 1 and 3 dpv revealed that the majority of DEGs were categorized into three major functional categories: biological process, cellular component, and molecular function (Fig 3C and 3D). These genes were found to be enriched in functions related to signaling transduction, immune response, and protein binding. Importantly, in the GO analysis, a significant number of DEGs related to molecular function were enriched in protein binding, including genes such as heat shock protein binding (Fig 3E). Kyoto Encyclopedia of Genes and Genomes (KEGG) analysis of 1 dpv versus 3 dpv showed that most DEGs enriched in MAPK signaling pathway, Endcocytosis, Spliceosome, Protein processing in endoplasmic reticulum and Ubiquitin mediated proteolysis (Figs 3F, S5E and S5F). Importantly, the largest numbers of DEGs enriched in the MAPK signaling pathway. According to the above results, the expression of genes related to heat shock protein binding and the MAPK signaling pathway in vaccinated fish at 1 and 3 dpv was evaluated (Fig 3G). qPCR results demonstrated that the expression levels of HSP70, JNK, p38, IL-6, and TRAF6 at 3 dpv were significantly higher than those at 1 dpv, indicating HSP70 and MAPK signaling pathway play a role in immune response of skin elicited by G3 protein.

### HSP70 interacts with SVCV G3 protein

**Fig 4:**
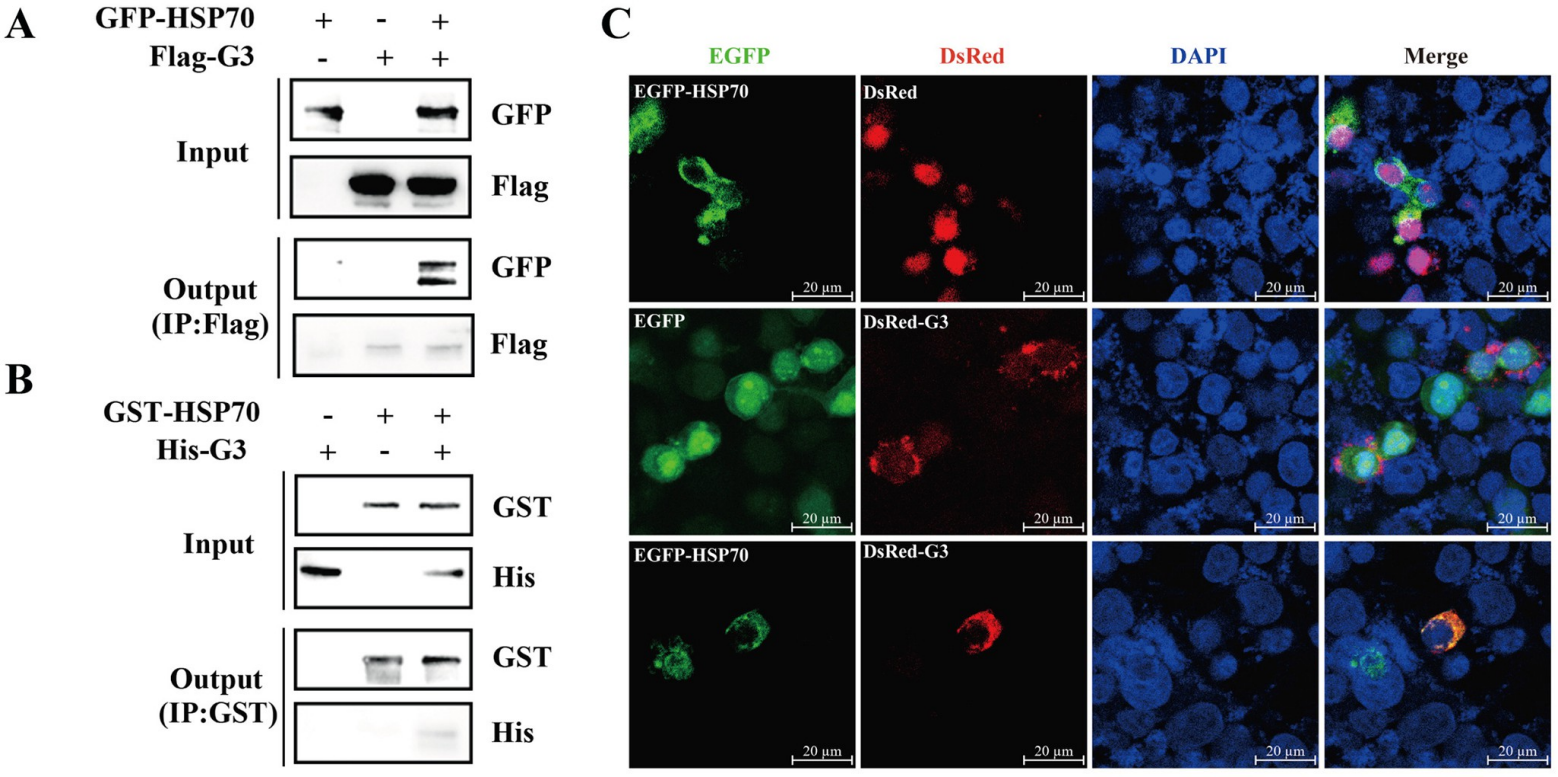
HSP70 interacts with SVCV G3 protein. (A) Co-immunoprecipitation were used to detect the interaction between HSP70 and G3. 293T cells were transfected with pEGFP-HSP70 and/or pcMV-Flag-G3. Then, the cells were collected 24 h post-transfection and subsequently subjected to co-immunoprecipitation assay using an anti-Flag antibody. (B) Pull down were used to detect the interaction between HSP70 and G3. His-G3 protein was incubated with GST-HSP70, and pull-down assays were conducted using anti-GST magnetic beads. The elution was subsequently analyzed by Western blotting. (C) Laser co localization were used to detect the interaction between HSP70 and G3. 293T cells were co-transfected with pEGFP-C1, pCMV-C-DsRed, pEGFP-HSP70, and pCMV-DsRed-G3, respectively. At 24 h post transfection, the cells were fixed, permeabilized, incubated with DAPI, and subsequently visualized using a confocal microscope. Scale bars, 20 μm.

To further analyze the role of HSP70 in G3-induced immune response, 293T cells were transfected with pEGFP-HSP70 and/or pcMV-Flag-G3, then coimmunoprecipitation (Co-IP) was performed to validate the interaction between HSP70 and G3 protein. Consequently, the presence of pEGFP-HSP70 was detected in the precipitate when 293T cell lysates co-expressing pcMV-Flag-G3 and pEGFP-HSP70 were subjected to immunoprecipitation using the Flag antibody, demonstrating the *in vivo* interaction of G3 protein with HSP70 protein (Fig 4A). Furthermore, pET-N-GST-HSP70 was constructed to obtained GST-HSP70 and purified with GST-tag Protein Purification Kit. Pull Down was used with purified G3 (His-G3), GST-HSP70, and anti-GST Magnetic Beads. After the mixture of His-G3 and GST-HSP70 was precipitated with anti-GST Magnetic Beads, His-G3 could be detected by anti-GST antibody, indicating that G3 protein can directly interact with HSP70 *in vitro* (Fig 4B). Subsequently, the subcellular localization of HSP70 and G3 was examined through confocal microscopy, revealing that EGFP-HSP70 (green fluorescent) and DsRed-G3 (red fluorescent) were localized in the cytoplasm and colocalized at various sites (yellow fluorescent) (Fig 4C). These findings provide clear evidence of the interaction between SVCV G3 protein and HSP70 protein.

### HSP70 mediates the immune response of G3 through the JNK/MAPK pathway

**Fig 5:**
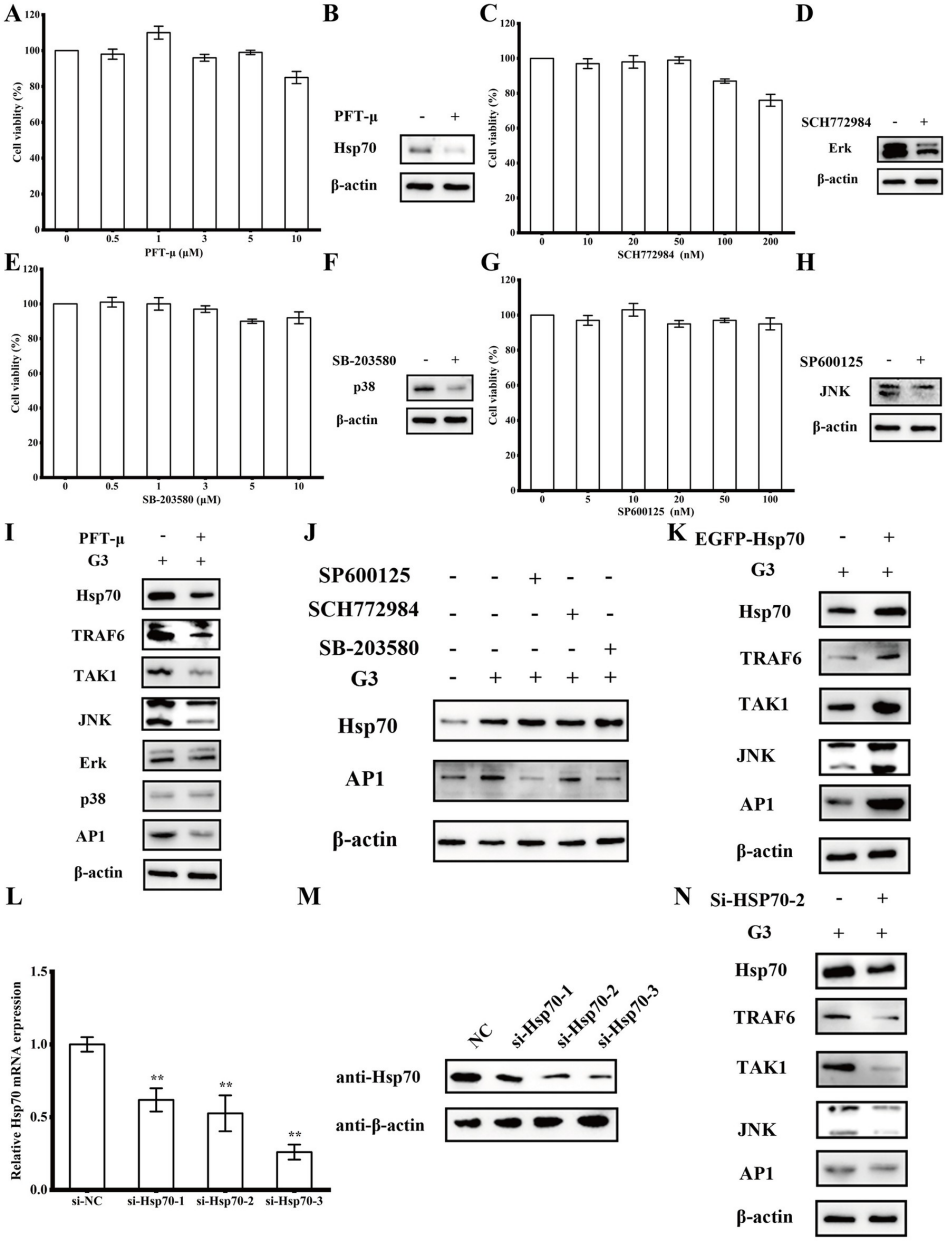
HSP70 mediates the immune response of G3 through the JNK/MAPK pathway. (A) The relative cell viability of ZF-4 cells after incubation with different concentrations of PFT-μ for 48 h. (B) The relative expression levels of HSP70 were examined by Western blotting after ZF-4 cells were cultured with 1 μM PFT-μ for 48 h. (C) The relative cell viability of ZF-4 cells after incubation with different concentrations of SCH772984 for 48 h. (D) The relative expression levels of ERK/MAPK were examined by Western blotting after ZF-4 cells were cultured with 10 nM SCH772984 for 48 h. (E) The relative cell viability of ZF-4 cells after incubation with different concentrations of SB-203580 for 48 h. (F) The relative expression levels of p38/MAPK were examined by Western blotting after ZF-4 cells were cultured with 1 μM SB-203580 for 48 h. (G) The relative cell viability of ZF-4 cells after incubation with different concentrations of SP600125 for 48 h. (H) The relative expression levels of JNK/MAPK were examined by Western blotting after ZF-4 cells were cultured with 10 nM SP600125 for 48 h. (I) The expression of indictors involved in HSP70-mediated MAPK signaling pathways upon treatment with HSP70 inhibitors was examined. Following 12-hour treatment with G3, ZF-4 cells were cultured with 1 μM PFT-μ for 48 hours. Subsequently, the cells were collected, and the protein expression levels were assessed via Western blotting. (J) The expression of HSP70-mediated AP1 upon treatment with HSP70 inhibitors was examined. Following 12-hour treatment with G3, ZF-4 cells were cultured with 1 μM SB-203580, 10 nM SCH772984, and 10 nM SP600125 for 48 h, respectively. Subsequently, the cells were collected, and the protein expression levels were assessed via Western blotting. (K) The expression of indictors involved in HSP70-mediated JNK/MAPK signaling pathways when HSP70 was overexpressed. Following 12-hour treatment with G3, ZF-4 cells were transfected with pEGFP-HSP70. Subsequently, the cells were collected, and the protein expression levels were assessed via Western blotting. (L) The RNA interference effect of HSP70 was detected by qPCR. ZF-4 cells were transfected with si-NC, si-Hsp70-1, si-Hsp70-2, or si-Hsp70-3. Subsequently, the cells were collected, and the mRNA expression levels of HSP70 in si-Hsp70-1 (0.61 ± 0.08), si-Hsp70-2 (0.53 ± 0.12), and si-Hsp70-3 (0.26 ± 0.05) groups were assessed via qPCR. (M) The RNA interference effect of HSP70 was detected by Western blotting. ZF-4 cells were transfected with si-NC, si-Hsp70-1, si-Hsp70-2, or si-Hsp70-3. Subsequently, the cells were collected, and the protein expression levels of HSP70 were assessed via Western blotting. (N) The expression of indictors involved in HSP70-mediated JNK/MAPK signaling pathways when HSP70 was knockdown. The P value for each study was calculated using one-way ANOVA. Statistical significance is indicated as follows: *P < 0.05, **P < 0.01. The data presented are representative of three independent experiments, with means ± standard error of the mean (SEM).

To further investigate the role of HSP70 in G3-induced immune response, HSP70 inhibitors PFT-μ was used and the toxicity to ZF-4 cells was analyzed firstly. Cell viability was not significantly affected by PFT-μ (99 ± 1.2%) at concentrations of up to 5 μM (Fig 5A), ZF-4 cells were cultured with 1 μM PFT-μ for 48 h can reduce the expression of HSP70 protein (Fig 5B). Transcriptome results showed that HSP70 and MAPK signaling pathway play a role in immune response, so we also used p38/MAPK inhibitors SB-203580, ERK/MAPK inhibitors SCH772984, and JNK/MAPK inhibitors SP600125. Cell viability assays showed negligible cytotoxic effects on ZF-4 cells at used concentrations, ZF-4 were cells cultured with 1 μM SB-203580 (100 ± 3.6%), 10 nM SCH772984 (97 ± 2.8%), and 10 nM SP600125 (103 ± 3.6%) for 48 h can reduce the expression of p38, ERK, and JNK protein (Fig 5C–5H).

To examine whether HSP70 activity was required for immune response of G3, treatment of ZF-4 cells with HSP70 inhibitors PFT-μ decreased G3-induced the expression of TRAF6 and TAK1, which subsequently compromised the activation of MAPK signaling, as indicated by the diminished expression of AP-1 (Fig 5I). But which groups of MAPKs plays the regulatory roles in G3-induced immune response still needs more investigate. Treatment of JNK/MAPK inhibitors SP600125 inhibitors PFT-μ decreased G3-induced the expression of AP-1, but no significant inhibition of AP-1 was observed in treatment with p38/MAPK inhibitors SB-203580 and ERK/MAPK inhibitors SCH772984 (Fig 5J), indicating that JNK/MAPK plays the regulatory roles in G3-induced immune response. HSP70 overexpression plasmid pEGFP-HSP70 were transfected to G3 pre-processed ZF-4 cells, results showed G3-induced the expression of TRAF6 and TAK1 were obvious enhanced, which subsequently facilitated the activation of JNK/MAPK signaling and the expression of AP-1 (Fig 5K). We constructed shRNA plasmid expressing three HSP70-specific siRNAs (si-HSP70-1, si-HSP70-2, and si-HSP70-3). Transfected three HSP70-specific siRNAs and si-NC (as control siRNA) into the cells, respectively, and the results showed that si-HSP70-3 caused a more pronounced elimination effect (Fig 5L and 5M). Then treatment of ZF-4 cells with si-HSP70-3 decreased G3-induced the expression of HSP70, JNK and AP-1 (Fig 5N). These data indicate that HSP70 can mediates the G3-induced immune response through the JNK to regulate MAPK pathway.

### HSP70 regulates G3-induced adaptive immune responses

**Fig 6:**
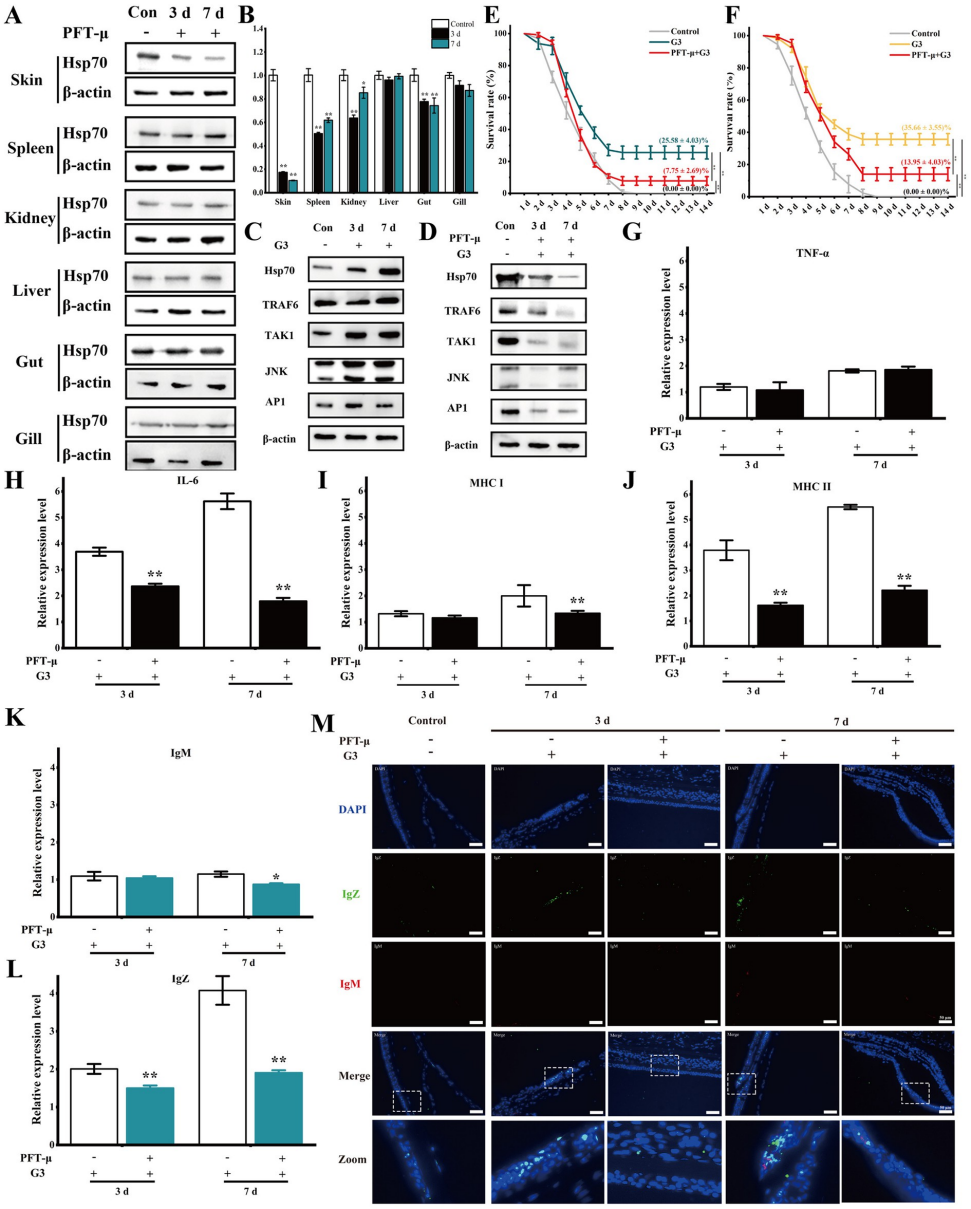
HSP70 regulates G3-induced immune responses. (A) The protein expression levels of HSP70 in different tissues of zebrafish after injection of HSP70 inhibitor. Following treatment with PFT-μ, different tissues of zebrafish at 3 and 7 d were collected, and the protein expression levels were detected by Western blotting. (B) Relative mRNA levels of *HSP70* in different tissues of zebrafish after injection of HSP70 inhibitor. Following treatment with PFT-μ, different tissues of zebrafish at 3 and 7 d were collected, and the mRNA expression levels were examined by qPCR. (C) The expression of HSP70-mediated MAPK signaling pathways in skin from vaccinated fish at 3 and 7 dpv. Following treatment with G3, skin from vaccinated fish at 3 and 7 dpv were collected, and the protein expression levels were detected by Western blotting. (D) The expression of indictors involved in HSP70-mediated MAPK signaling pathways in skin from vaccinated fish which treated with HSP70 inhibitors. After treatment with G3, vaccinated fish were injected with PFT-μ. Subsequently, skin from vaccinated fish at 3 and 7 dpv were collected, and the protein expression levels were detected by Western blotting. (E) After 3 days post-vaccination, survival rate of vaccinated fish challenged with SVCV. (F) After 7 days post-vaccination, survival rate of vaccinated fish challenged with SVCV. (G) The relative protein expression levels of TNF-α in skin of vaccinated fish with HSP70-inhibited were analyzed at various time points using ELISA. The expression level of TNF-α in the skin was 1.08 ± 0.3 at 3 dpv and 1.86 ± 0.12 at 7 dpv in HSP70-inhibited groups. (H) The relative protein expression levels of IL-6 in skin of vaccinated fish with HSP70-inhibited were analyzed at various time points using ELISA. The expression level of IL-6 in the skin was 2.36 ± 0.1 at 3 dpv and 1.79 ± 0.13 at 7 dpv in HSP70-inhibited groups. (I) The relative protein expression levels of MHC I in skin of vaccinated fish with HSP70-inhibited were analyzed at various time points using ELISA. The expression level of MHC I in the skin was 1.16 ± 0.09 at 3 dpv and 1.33 ± 0.09 at 7 dpv in HSP70-inhibited groups. (J) The relative protein expression levels of MHC II in skin of vaccinated fish with HSP70-inhibited were analyzed at various time points using ELISA. The expression level of MHC II in the skin was 1.61 ± 0.1 at 3 dpv and 2.21 ± 0.18 at 7 dpv in HSP70-inhibited groups. (K) The relative protein expression levels of IgM in skin of vaccinated fish with HSP70-inhibited were analyzed at various time points using ELISA. The expression level of IgM in the skin was 1.04 ± 0.05 at 3 dpv and 0.87 ± 0.03 at 7 dpv in HSP70-inhibited groups. (L) The relative protein expression levels of IgZ in skin of vaccinated fish with HSP70-inhibited were analyzed at various time points using ELISA. The expression level of IgZ in the skin was 1.5 ± 0.07 at 3 dpv and 1.91 ± 0.06 at 7 dpv in HSP70-inhibited groups. (M) Representative immunofluorescence images for IgZ (green) and IgM (red) of skin from vaccinated fish on days 3 and 7 after immunization. Nuclei were stained with DAPI (blue). Scale bars, 50 μm. The P value for each study was calculated using one-way ANOVA. Statistical significance is indicated as follows: *P < 0.05, **P < 0.01. The data presented are representative of three independent experiments, with means ± standard error of the mean (SEM).

After injecting inhibitors into zebrafish, we detected the mRNA and protein expression levels of HSP70 in different tissues, the results showed that the mRNA and protein expression levels of HSP70 in skin tissues were significantly reduced (Fig 6A and 6B). To further confirm the regulatory role of HSP70 in teleost adaptive immunity *in vivo*, we found treatment of zebrafish with HSP70 inhibitors PFT-μ decreased G3-induced the expression of JNK/MAPK and AP-1, and MAPK signaling pathways in skin of HSP70-inhibited zebrafish were significantly inhibited (Fig 6C and 6D). Additionally, the loss of effector function due to HSP70 inhibition markedly increased the mortality of fish at 3 dpv and 7 dpv following SVCV infection compared to the G3 group (Fig 6E and 6F). After 3 days post-vaccination, survival rate of vaccinated fish challenged with SVCV, the survival rate of the G3 immune group after HSP70 inhibition was 7.75 ± 2.69%, while that of the G3 group was 25.58 ± 4.03%. After 7 days post-vaccination, survival rate of vaccinated fish challenged with SVCV, the survival rate of the G3 immune group after HSP70 inhibition was 13.95 ± 4.03%, while that of the G3 group was 35.66 ± 3.55%. Moreover, survival analyses used log-rank test indicated that there were significant differences between 3 dpv or 7 dpv and the control group (S6 Fig). Using HSP70-inhibited zebrafish, we further demonstrated the pivotal role of HSP70 in G3-induced immune responses. Compared to vaccinated zebrafish, the expression of MHC-I, MHC-II, TNF-α, and IL-6 was significantly inhibited in the skin of vaccinated HSP70-inhibited zebrafish (Fig 6G–6J). Moreover, the expression of IgZ and IgM in the skin of vaccinated HSP70-inhibited zebrafish was significantly lower than that in vaccinated zebrafish (Fig 6K–6M). These findings collectively indicate the indispensable role of HSP70 in eliciting immune responses in zebrafish.

## Discussion

Skin serves as a physical and immune barrier, separating the internal and external environments to shield organisms from pathogens [33,34]. Therefore, vaccination through the skin is widely considered to be the most effective strategy for triggering a mucosal immune response [35]. The surface of bony fish skin adapted to aquatic environments consists of an epidermis and dermis, and is enveloped by mucus, which is an integral part of their defense mechanism against pathogens [32]. This contrasts with the skin of mammals, which lacks a mucous surface but has a corneum outermost layer that primarily serves as a physical barrier to protect the skin [36]. Teleost skin has been shown to contain skin-associated lymphoid tissue (SALT) that consists of multiple immune cells including B cells, T cells, granulocytes, macrophages, and Langerhans-like cells, recognition of pathogens stimulates the production of various cytokines that activate the adaptive immune system through antigen presentation and cytokine stimulation [36,37].

More and more studies support that immersion vaccination stimulates mucosal immune responses in the skin, prompting the body to mount an immune response and providing robust protection against pathogens [38,39]. In this study, zebrafish were immersion vaccine with G3 for 6 h, and through *in vivo* imaging observation, we observed a gradual increase in skin uptake of the vaccine with prolonged immune duration. Even at 12 h and 24 h after immunization, when the zebrafish had been transferred to clean water, residual fluorescence was still detectable in the skin. In addition, large amounts of fluorescence were observed in the epidermal layer at 6 h after immunization, while more pronounced fluorescence was observed in the dermis and muscles at 12 h and 24 h after immunization. As time progresses, the uptake of the vaccine by the epidermis gradually metabolizes to the dermis and muscles, revealing the vital role of skin uptake of the vaccine during immersion immunization process. In SVCV infection, 26% of the immune protection effect can be produced after 3 dpv. These results suggest that the skin takes in a large amount of vaccine very soon after immunization, stimulating the body to produce immune response even at 3 dpv. Specific antibodies were detected in both systemic and mucosal immunity after exposure to pathogens, and the skin was the earliest to detect specific antibodies, indicating that skin plays a key role in the early stage of immunity [39]. Our work showed that skin is involved in the uptake and metabolism of vaccines in the early stages of immunity, and induces immune responses shortly after contact with the vaccine. Therefore, the promotion of skin and mucosal barrier function is of great significance for the sustainable disease resistance of aquaculture.

In teleost fish, the B cells and immunoglobulins (Ig) within the teleost mucosa associated lymphoid tissues (MALTs) constitute the main humoral adaptive immune response [40–42], and three classes of Ig have been identified: IgM, IgD, and IgZ/T [43]. IgM, widely present in teleost fish plasma, plays a crucial role in adaptive immune responses, such as antibody-dependent cell-mediated cytotoxicity and complement activation [44–46]. Although IgD is widespread in many vertebrate classes, its function remains poorly understood [47,48]. The immunoglobulin IgT/Z is produced only in teleost fish which was first identified in rainbow trout (IgT) and zebrafish (IgZ), has been demonstrated to play a specialized role in mucosal immunity akin to that of mammalian secretory IgA (sIgA) [19,49,50]. Low concentrations of IgT/Z are detectable in the serum of rainbow trout, with levels significantly lower than those of IgM, whereas IgT/Z concentrations in the gut are substantially higher than in the serum [51]. IgT^+^ B cells have been identified in the skin of rainbow trout, and bacterial infection has been shown to enhance the proliferation of IgT^+^ B cells in the skin and the secretion of IgT, while the number of skin IgM^+^ B cells remains relatively unchanged [52]. In this study, a significant increase in IgZ+ B cells in zebrafish skin was observed at 3dpv, with even higher levels evident at 7 dpv. Conversely, IgM^+^ B cells were observed only in small numbers. Furthermore, significant cell proliferation was detected in the skin at 7 dpv, accompanied by a marked increase in IgZ antibody secretion, suggesting that vaccination significantly stimulates the proliferation of IgZ^+^ B cells in the skin post-immunization. Therefore, skin should be the prime targets for studying molecular mechanisms of adaptive immunity. Understanding how vaccination stimulates early adaptive immune responses is critical for animal and human health.

In order to reveal the early immune regulation mechanism induced by skin in zebrafish, high-throughput transcriptome sequencing was employed to analyze the skin of zebrafish after vaccine immunization. Our results indicate that HSP70 and MAPK signaling pathway play a role in immune response of skin elicited by G3 protein. As a molecular chaperone with a wide range of functions, HSP70 plays an important role in cell signaling and the immune system, presents immune and inflammatory cytokines, and plays a crucial role in regulating cellular differentiation, survival, and apoptosis [53–55]. HSP70 protein, utilized as a vaccine platform constructed from tumor cell membranes with immune accompaniment, exhibits favorable biosafety profiles and demonstrates significant reductions in tumorigenicity alongside inhibition of tumor growth. Collaboration between antigens and immune-associated proteins can promote the uptake and cross-presentation of cancer antigens, thereby enhancing cancer-specific immunogenicity [56]. Recombinant LcrV-HSP70, designed for *Yersinia pestis* and *M*. *tuberculosis*, induced significant expression of IFN-γ and TNF-α in immune mice, providing 100% protection against plague and leading to a marked decrease in the colony-forming units (CFU/mL) of *Yersinia* and *Yersinia pseudotuberculosis* in blood and spleen [57]. HSP70 interacts with Toll-like receptor 2 (TLR2) to activate myeloid-derived suppressor cells (MDSCs), thereby stimulating the nuclear factor-Kappa B signaling pathway, and activating JAK2 (Janus Kinase)/STAT3 signaling pathway through IL-6 autocrine secretion [58]. MAPK family has been shown to regulate adaptive immune responses by regulating inflammatory and anti-inflammatory cytokine production, antigen presentation, cytokine synthesis, and cell proliferation, which including the extracellular signal-regulated kinases (ERK), the c-Jun amino-terminal kinases (JNK) and the p38-MAPK [59,60]. Hsp70 has been shown to interact with the MK2 which substrate of p38/MAPK, knockdown of Hsp70 can lead to downregulation of MK2 and p38/MAPK in intact muscles cells, and overexpression of p38/MAPK can restore the damage caused by HSP70-deficient cells [61]. Inhibition of JNK1/2 can decrease the expression of HSP70 in human nasal epithelial cells (HNEpCs), while activation of JNK/MAPK signaling pathway can increase the expression of HSP70 in HNEpCs [62].

As expected, our further results suggest that G3 protein interacts with HSP70 to regulate immune responses through JNK to regulate MAPK pathway, and that knocking down HSP70 inhibits JNK/MAPK and downstream pathway expression. The G protein of SVCV is the only viral protein that exists on the surface of the virus particle and forms a trimer, binds to the cell receptor to induce viral endocytosis, is the target of protective neutralizing antibodies, and is a commonly used antigenic protein of rhabdoviruses [63,64]. Both DNA vaccine [65] and subunit vaccine [66] constructed with SVCV G can induce strong immune response and provide effective protection against SVCV infection. G proteins have been shown to bind to host proteins and play an important role in virus-host interaction or regulation of host immune response [38,67]. A host protein (14-3-3β/α-A) from FHM cell was identified can protein interacted with SVCV-G protein, the mRNA and protein expression level were downregulated due to SVCV infection which may play an essential role in the early stages of SVCV life cycle [31]. The mucosal vaccine (LSG-TDH) constructed from SVCV G is effectively absorbed by mucosal tissues by immersion vaccination and activates antigen presenting cells (APCs) at the local mucosal site, which then induces a powerful local mucosal and systemic immune response [38]. In addition, G protein mediates these immune effects by interacting with Toll-like receptor 2 (TLR2) and activating the downstream NF-κB signaling pathway [68]. In our study, immunized HSP70-inhibited zebrafish could not provide effective protection against SVCV infection, the expression of IgZ and cytokines in the skin was significantly lower than that in vaccinated zebrafish. HSP70 has been shown to have immune-stimulating effects, when combined with an antigen (immobilization antigen, iAg) against *Cryptocaryon irritans* infection, HSP70 can upregulate expression of T cell markers CD8α and CD4, TNF-α, MHC-I and MHC-II in grouper. In addition, significantly high levels of iAg-specific antibodies and non-specific CD8^+^ skin leucocytes were detected in the skin [69]. The role of HSP70 in immune response is still relatively little studied, especially how to participate in and regulate mucosal immunity and adaptive immune response remains to be explored.

Taken together, we reported that the important role of HSP70 in early skin immune responses. Glycoprotein interacts with HSP70 to regulate MAPK through JNK, thereby modulating mucosal and systemic immune responses. Further investigations demonstrated that HSP70-inhibited zebrafish showed a significant reduction in survival rate after viral challenge and the production of IgM and IgZ in the skin. Our research provides important insight for revealing the skin immune mechanisms of vertebrates and understanding the development of the immune system.

## Materials and methods

### Ethics statement

Healthy zebrafish (Danio rerio) (0.8 ± 0.2 g) were purchased from a local market in Yangling (Shaanxi, China). After the carp was purchased, it was tested without SVCV infection, and it was cultured in the laboratory for three weeks (water temperature as 20–22°C, fed with pelleted carp food thrice a day) before being used in the experiment. All activities were carried out following the guidelines outlined in the Guide for the Care and Use of Animals at Northwest A&F University. The animal use protocol has been reviewed and approved by the Animal Ethical and Welfare Committee, Northwest A&F University, China (Approval No. DK2021001).

### Cells, virus, plasmids, antibodies, and regents

ZF4 cells (Zebrafish embryo fibroblast-like) and HEK 293T cells (Human embryonic kidney 293T) were stored in our laboratory. ZF-4 cells were cultivated in F12 medium (Hyclone, USA), HEK 293T cells were grown in DMEM medium (Hyclone, USA), 10% FBS (ZETA LIFE, USA), 100 U/mL penicillin (Hyclone, USA), 100 μg/mL streptomycin (Hyclone, USA) were supplemented in the medium. Zebrafish macrophages were isolated from head kidney used Fish Monocyte Isolation Kit (Solarbio, China) as previously described [10,37]. The head kidney tissue was minced in F12 medium with 10% FBS, 100 U/mL penicillin, and 100 μg/mL streptomycin, passed through a 100 μm filter (Solarbio, China) to obtain a single-cell suspension, and then processed using Percoll density gradient separation. Following centrifugation at 500 g for 25 minutes, the cells were resuspended in F12 medium and cultured at 25°C for 4 hours. Non-adherent cells were removed, leaving the adherent cells identified as monocytes/macrophages.

SVCV was preserved in our lab and propagated in ZF-4 cells grown in M199 medium (Hyclone, USA) with 10% FBS. Virus titers were determined by a previously reported method [70] and were given as plaque-forming units.

The plasmids pET32a and pEGFP-C1 was preserved in our laboratory. The plasmids pCMV-C-DsRed, pET-N-GST, and pCMV-C-Flag were obtained from Beyotime (Shanghai, China). The plasmid pLKO.1-EGFP-Puro was obtained from Miaoling Biology (Wuhan, China). The plasmids pET32a-His-G3, pCMV-DsRed-G3, and pCMV-Flag-G3 encoding SVCV glycoprotein (G^251-381^, S1 Table), pEGFP-HSP70 and pET-GST- HSP70 encoding HSP70 gene of zebrafish were constructed in our laboratory. The plasmids pLKO.1-si-HSP70-1, pLKO.1-si-HSP70-2, and pLKO.1-si-HSP70-3 were constructed in our laboratory, the primers and their sequences are shown in S2 Table.

The mouse anti-β-actin, anti-GFP, anti-Flag, and anti-GST antibodies were purchased from Proteintech (Wuhan, China). The rabbit anti-HSP70 and anti-Krt8 antibodies were purchased from Proteintech (Wuhan, China). The rabbit anti-TRAF6, anti-TAK1, anti-JNK, anti-ERK, anti-p38, anti-AP1 antibodies were purchased from Beyotime (Shanghai, China). The mouse anti-zebrafish IgM antibody was purchased from Aquatic Diagnostics Ltd (Stirling, Scotland). The rabbit anti-zebrafish IgZ antibody was preserved in our lab which performed according to our previous study [38,68]. The secondary antibodies HRP-anti-rabbit IgG, HRP-anti-mouse IgG, Cy3-anti-mouse IgG, Cy3-anti-rabbit IgG, FITC-anti-mouse IgG, and FITC-anti-rabbit IgG antibodies were purchased from Proteintech (Wuhan, China).

PFT-μ (HSP70 inhibitor, CAS: 64984-31-2), SB-203580 (p38 inhibitor, CAS: 152121-47-6), SCH772984 (ERK inhibitor, CAS: 942183-80-4), and SP600125(JNK inhibitor, CAS: 129-56-6) were purchased from MedChemExpress (New Jersey, USA).

### Immersion vaccination and SVCV challenge

The purified recombinant G3 protein was preserved in our laboratory. Zebrafish were tested without SVCV infection by qPCR. SVCV-free zebrafish were randomly divided into control and G3 groups (75 fish per group) at 30 mg/L, then vaccinated for 6 h by bath immunization. After vaccination, transferred all fish to the different tanks with fresh water. At 3 d, 7 d, 14 d, and 28 d post-vaccination, fish (43 fish per group, every group made three replicates) from each group were taken to exposed to a dilution of SVCV (1 × 10^7^ pfu/mL) for in a volume of 2 L, respectively. Dead fish are collected and recorded daily.

### Cellular uptake, cell viability analysis, and in vivo fluorescence imaging

To track the kinetics of G3, G3 protein was labeled with FITC to obtain the FITC-G3 according to our previous study [38,68]. FITC-G3 at 30 μg/mL were added in 12-well plates with macrophages for 24 h. Furthermore, treated macrophages were stained with DAPI (Beyotime, China). After thorough washing with PBS, the cells were observed by fluorescence microscopy (LECIA, DM6 B, Germany). Cell viability was determined by the 3-(4,5-dimethyl-2-thiazolyl)-2,5-diphenyl-2H-tetrazolium bromide (MTT) assay (Beyotime, China) following the standard protocol.

Zebrafish (n = 30) treated for 6 h with FITC-G3 at a 30 mg/L. Afterwards, the treated fish were transferred to clean water. After that, the fish and skin were isolated to observe by the Multimodal animal *in vivo* imaging system (Guangzhou Biolight Biotechnology Co., Ltd, AniView 100, China) at different time points (0, 1, 3, 6, 12, 24, 48, 72, 120, 168, 216, and 336 h) post-vaccination.

### Histology, light microscopy, and immunofluorescence microscopy studies

To detect the histological structure and mucus cells in skin of vaccination fish, the sections of skin were stained with H&E and Alcian blue (AB). To detect the distribution of G3 in skin, frozen slices are made and stained with DAPI (Beyotime, China). To detect the existence of IgZ and IgM in skin, frozen slices are made, then mouse anti-zebrafish IgM antibody and rabbit anti-zebrafish IgZ antibody were used to assess the IgT^+^ and IgM^+^ B cells. Krt-8 antibody was used to label epidermal cells. Cy3-anti-mouse IgG, Cy3-anti-rabbit IgG, FITC-anti-mouse IgG, and FITC-anti-rabbit IgG were used as second antibodies. The frozen slices were finally stained by DAPI and sealed. All images were gained and analyzed by observed by fluorescence microscopy (LECIA, DM6 B, Germany).

### RNA isolation and quantitative real-time PCR (qPCR) analysis

The spleen tissues were collected in Trizol reagent (Takara, China), and extracted total RNA by the instruction of the manufacturer. Further, cDNA synthesis used by HiScript Q RT SuperMix for qPCR kit (Vazyme, China) from total RNA by the manufacturer’s instructions. The obtained cDNAs were used to detect the viral load (SVCV-N gene) and expression of skin immune related genes by qPCR. qPCR was performed with CFX96 Real-Time PCR Detection System (Bio-Rad, USA) using AceQ qPCR SYBR Green Master Mix (Vazyme, China) and calculated by 2^-ΔΔCt^ method [71]. The primers of detected genes are listed in S3 Table.

### Enzyme linked immunosorbent assay (ELISA) analysis

To detect the protein expression of IgZ and IgM, samples was added to the ELISA plate at 4°C overnight to make it coated on the ELISA plate. After washing with PBST for three times, the sample was added with 2% BSA (Solarbio, China) and closed at 37°C for 1 h. After washing PBST for three times, mouse anti-zebrafish IgM antibody and rabbit anti-zebrafish IgZ antibody were added and incubated at 37°C for 2 h. After washing PBST for three times, HRP labeled IgG secondary antibody was added. Then TMB chromogen solution (Solarbio, China) was added, and the absorbance at 450 nm was measured using a precision microplate reader (Bio Tek, USA). Enzymes activities (SOD (superoxide dismutase) activity, AKP (alkaline phosphatase) activity and ACP (acid phosphatase) activity assay) and C3 (complement) content were analysed by using a measurement kit (Jiancheng Bioengineering Research Institute, China) according to the manufacturer’s instructions. The protein expressions of IL-6, TNF-α, MHC-I, and MHC-II were analyzed by corresponding ELISA kits (Renjiebio, China) according to the manufacturer’s instructions.

### RNA-seq libraries and differential expression analysis

Zebrafish skin at 1 dpv, 3 dpv, and a control group that was not treated (each group contain three samples, with five fish per sample) were sampled and used for the RNA-Seq libraries construction. We performed the 2×150 bp paired-end sequencing (PE150) on an Illumina Novaseq 6000 (LCBio Technology CO., Ltd., Hangzhou, China) following the vendor’s recommended protocol. After filtering the reads by Cutadapt (https://cutadapt.readthedocs.io/en/stable/, version:cutadapt-1.9), high-quality sequencing data was obtained also known as clean data. Then sequence quality was verified using FastQC (http://www.bioinformatics.babraham.ac.uk/projects/fastqc/, 0.11.9) including the Q20, Q30 and GC-content of the clean data. The average clean data of each sample reached 6.32 Gb and the percentage of Q30 was more than 97.43%. After that, a total of 126,349,098 cleaned reads were produced. We aligned reads of all samples to the < *Danio rerio* > reference genome using HISAT2 (https://daehwankimlab.github.io/hisat2/, version:hisat2-2.2.1) package, which initially remove a portion of the reads based on quality information accompanying each read and then maps the reads to the reference genome. Then the gene expression quantitative analysis, gene difference analysis, enrichment analysis and other analyses are carried out. Genes differential expression analysis was performed by DESeq2 software between two different groups (and by edgeR between two samples). The genes with the parameter of false discovery rate (FDR) below 0.05 and absolute fold change ≥ 2 were considered differentially expressed genes (DEGs). Differentially expressed genes were then subjected to enrichment analysis of GO functions and KEGG pathways. Bioinformatic analysis was performed using the OmicStudio tools at https://www.omicstudio.cn/tool.

### Plasmid transfection, coimmunoprecipitation, and pull down

The 293T cells were inoculated in the six-well plate in advance so that the cell density was about 80%. The recombinant plasmid was then transfected with Lipo8000 Transfection Reagent (Beyotime, China) according to the protocol provided by the manufacturer. For coimmunoprecipitation, the cells after transfection with plasmids were washed three times with PBS, and the cells were slowly lysed with Cell lysis buffer for Western and IP (Beyotime, China). Centrifuge at 4°C 1000g for 3 minutes and collect the supernatant. Subsequently, the supernatant and anti-FLAG antibody were incubated at room temperature for 2 hours. Incubate overnight with Protein A+G Magnetic Beads (Beyotime, China) at 4°C to precipitate the interacting complex. The elution sample was used for Western blotting.

For pull down, plasmid pET-N-HSP70 was transformed into *E*. *coli* BL21 cells (Tsingke, China). The expression of recombinant GST-HSP70 protein was induced by isopropyl-β-D-thiogalactose-pyranoside (IPTG, Solarbio, China). After the cells are destroyed by ultrasound, GST-tag protein purification kit (Beyotime, China) was used to obtain purified GST-HSP70 proteins. His-G3 protein (0.5 mg) and GST-HSP70 (0.5 mg) were incubated at 4°C for 30 min, then BeyoMag Anti-GST Magnetic Beads (Beyotime, China) was added to the mixture and incubated at 4°C overnight. The elution sample was analyzed by SDS-PAGE and Western blotting.

### Laser confocal scanning microscopy assays

The 293T cells were inoculated in a 20-mm dish, so that culture results the cell density was about 80%. The recombinant plasmid was then transfected with Lipo8000 Transfection Reagent, then the cells were fixed with 4% PFA for 1 h. After washing with PBS, the cells were stained by DAPI. Images were obtained with Laser Scanning Confocal Microscopy (Carl Zeiss, LSM980, Germany).

### Western blot

The sample was boiled for 15 min after adding the sample buffer, then separated by SDS-PAGE and transferred to PVDF membrane (Solarbio, China). The membranes were blocked with QuickBlock Blocking Buffer (Beyotime, China) for 1 h at room temperature, and then the primary antibody was incubated at 4°C overnight. After washed with TBST buffer (25 mM Tris-HCl, 150 mM NaCl, 0.1% Tween 20, pH 7.5) for three times, secondary antibodies (HRP-anti-Mouse IgG or HRP-anti-Rabbit IgG) was incubated at 37°C for 1.5 h. After washed with TBST buffer for three times, enhanced chemiluminescence (ECL) reagents (Solarbio, China) was used for immunodetection.

### Statistical analysis

All statistical analyses were performed using SPSS 18.0 software (SPSS Inc., USA). To evaluate exposure data, the *P*-value was analyzed by Student’s t test or one-way ANOVA after normalization. Log-rank test was performed for analyzing statistical significance of survival curve analysis. Data are expressed as mean ± analysis of variance. In all cases, the differences were considered significant at P < 0.05 and extremely significant at P < 0.01.

## Supporting information

S1 Fig Cellular uptake of G3 by macrophage. (A) SDS-PAGE analysis was performed on the purified His-G3 recombinant protein. Lane M, standard protein marker. Lane 1, insoluble lysates from *E*.*coli* BL21 strain transformed with pET32a-G3 and induced by IPTG. Lane 2, insoluble lysates from *E*.*coli* BL21 strain transformed with pET32a-G3 that was uninduced. Lane 3, the purified recombinant His-G3 protein. (B) The relative cell viability of macrophages following incubation with G3 for different durations was assessed. After co culturing macrophages with G3 for 12 hours, there was no significant difference in cell viability (99 ± 2.1%) compared to the control group. (C) *In vitro* cellular uptake of G3 by macrophage was demonstrated through representative immunofluorescence images of macrophages following incubation with G3. The G3 were labeled with FITC (green channel), while the nuclei nucleus were labeled with DAPI (blue channel).
(TIF)

S2 Fig Survival curve analysis (Kaplan-Meier) for virus challenge after G3 immunization, and compared by log-rank analysis. (A) After 3 days post-vaccination, the survival curve of immunized fish was analyzed for virus challenge. (B) After 7 days post-vaccination, the survival curve of immunized fish was analyzed for virus challenge. (C) After 14 days post-vaccination, the survival curve of immunized fish was analyzed for virus challenge. (D) After 28 days post-vaccination, the survival curve of immunized fish was analyzed for virus challenge. Survival curve analysis for each study was calculated using Kaplan-Meier method. Combine three replicates (43 fish per replicate) into one group for analysis. Log-rank test was performed for analyzing statistical significance, *P < 0.05, **P < 0.01.
(TIF)

S3 Fig Strong immunoprotection were elicited by G3 following immersion vaccination. (A) The viral load in the spleen of vaccinated fish was quantified at different times post-vaccination using qPCR. (B) The viral load in the skin of vaccinated fish was quantified at different times post-vaccination using qPCR. (C) The ACP activities in the skin of vaccinated fish were measured at different times post-vaccination using ELISA. The ACP activities were significantly higher in G3 groups at 3 dpv (2.16 ± 0.06) and 7 dpv (2.5 ± 0.02) compared to the control group. (D) The AKP activities in the skin of vaccinated fish were measured at different times post-vaccination using ELISA. The AKP activities were significantly higher in G3 groups at 3 dpv (1.86 ± 0.06) and 7 dpv (2.0 ± 0.13) compared to the control group. (E) The SOD activities in the skin of vaccinated fish were measured at different times post-vaccination using ELISA. The SOD activities were significantly higher in G3 groups at 7 dpv (87.91 ± 1.43) compared to the control group. (F) The C3 content in the skin of vaccinated fish were measured at different times post-vaccination using ELISA. The C3 content were significantly higher in G3 groups at 3 dpv (1.15 ± 0.04) and 7 dpv (1.32 ± 0.02) compared to the control group. (G) Histological examination was performed using H&E staining of skin from vaccinated fish at 14 dpv. Scale bars, 50 μm. The P value for each study was calculated using Student’s t tests and one-way ANOVA. Statistical significance is indicated as follows: *P < 0.05, **P < 0.01. The data presented are representative of three independent experiments, with means ± standard error of the mean (SEM).
(TIF)

S4 Fig G3 immunization promotes cell proliferation and B cell immune response. (A) Immunofluorescence analysis was performed to assess cell proliferation through EdU incorporation in the skin of vaccinated fish. EdU (red) was used to detect the cell proliferation in the skin tissue sections, Krt-8 (green) is used to label epidermal cells, nuclei were stained with DAPI (blue). Scale bars, 50 μm. (B) Representative immunofluorescence images for IgM (green) of skin from vaccinated fish on days 3 and 7 after immunization. Krt-8 (red) is used to label epidermal cells. Nuclei were stained with DAPI (blue). Scale bars, 50 μm. (C) Representative immunofluorescence images for IgZ (green) of skin from vaccinated fish on days 3 and 7 after immunization. Krt-8 (red) is used to label epidermal cells. Nuclei were stained with DAPI (blue). Scale bars, 50 μm.
(TIF)

S5 Fig DEGs in zebrafish skin after immersion vaccinated compared with control group. (A) Validation of transcriptomic data of upregulated genes at 1 dpv by qPCR. (B) Validation of transcriptomic data of downregulated genes at 1 dpv by qPCR. (C) Validation of transcriptomic data of upregulated genes at 3 dpv by qPCR. (D) Validation of transcriptomic data of downregulated genes at 3 dpv by qPCR. (E) The DEGs enrichment KEGG pathway analysis at 1 dpv compared with control group. (F) The DEGs enrichment KEGG pathway analysis at 3 dpv compared with control group. Data of qPCR are representative of three different independent experiment and shown as means ± SEM.
(TIF)

S6 Fig Survival curve analysis (Kaplan-Meier) for SVCV challenge after vaccinating zebrafish with HSP70-inhibited, and compared by log-rank analysis. (A) After 3 days post-vaccination, the survival curve of vaccinated fish challenged with SVCV. (B) After 7 days post-vaccination, the survival curve of vaccinated fish challenged with SVCV. Survival analysis for each study was calculated using Kaplan-Meier method. Combine three replicates (43 fish per replicate) into one group for analysis. Log-rank test was performed for analyzing statistical significance, *P < 0.05, **P < 0.01.
(TIF)

S1 Table Amino acid sequences of G3 (glycoprotein, G^251-381^). (DOCX)

S2 Table Primers used for shRNA plasmid expressing HSP70-specific siRNAs. (DOCX)

S3 Table Primers used for the analysis of mRNA expression by qPCR. (DOCX)
