# Supplementary figures and images for: Skin as outermost immune organ of vertebrates that elicits robust early immune responses after immunization with glycoprotein of spring viraemia of carp virus
Source: PLoS Pathog. 2024 Dec 9;20(12):e1012744. doi: 10.1371/journal.ppat.1012744 (PMC11627376; doi:10.1371/journal.ppat.1012744)

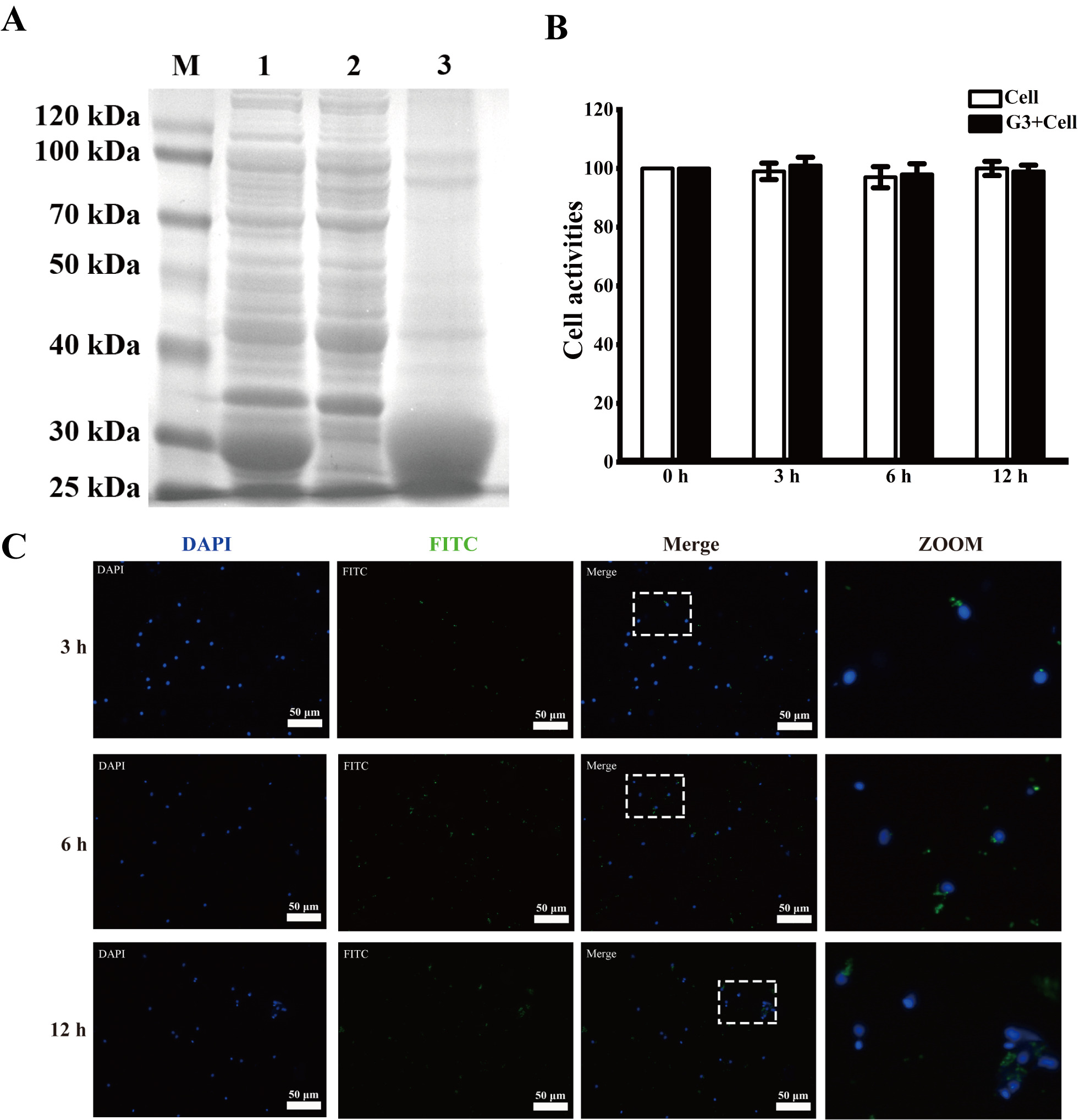

Supplement: S1 Fig — (A) SDS-PAGE analysis was performed on the purified His-G3 recombinant protein. Lane M, standard protein marker. Lane 1, insoluble lysates from E.coli BL21 strain transformed with pET32a-G3 and induced by IPTG. Lane 2, insoluble lysates from E.coli BL21 strain transformed with pET32a-G3 that was uninduced. Lane 3, the purified recombinant His-G3 protein. (B) The relative cell viability of macrophages following incubation with G3 for different durations was assessed. After co culturing macrophages with G3 for 12 hours, there was no significant difference in cell viability (99 ± 2.1%) compared to the control group. (C) In vitro cellular uptake of G3 by macrophage was demonstrated through representative immunofluorescence images of macrophages following incubation with G3. The G3 were labeled with FITC (green channel), while the nuclei nucleus were labeled with DAPI (blue channel). (TIF) [file ppat.1012744.s001.tif]

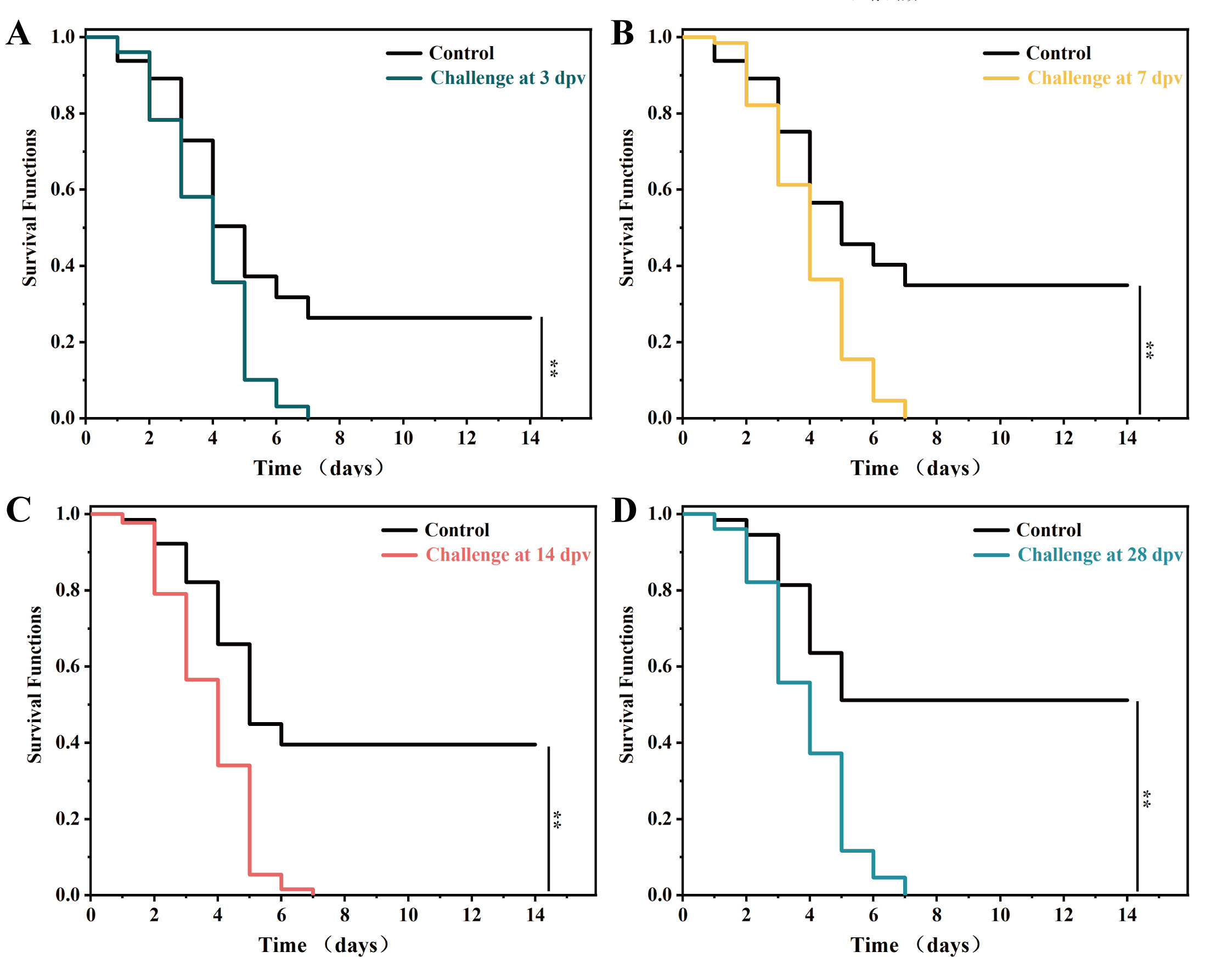

Supplement: S2 Fig — (A) After 3 days post-vaccination, the survival curve of immunized fish was analyzed for virus challenge. (B) After 7 days post-vaccination, the survival curve of immunized fish was analyzed for virus challenge. (C) After 14 days post-vaccination, the survival curve of immunized fish was analyzed for virus challenge. (D) After 28 days post-vaccination, the survival curve of immunized fish was analyzed for virus challenge. Survival curve analysis for each study was calculated using Kaplan-Meier method. Combine three replicates (43 fish per replicate) into one group for analysis. Log-rank test was performed for analyzing statistical significance, *P < 0.05, **P < 0.01. (TIF) [file ppat.1012744.s002.tif]

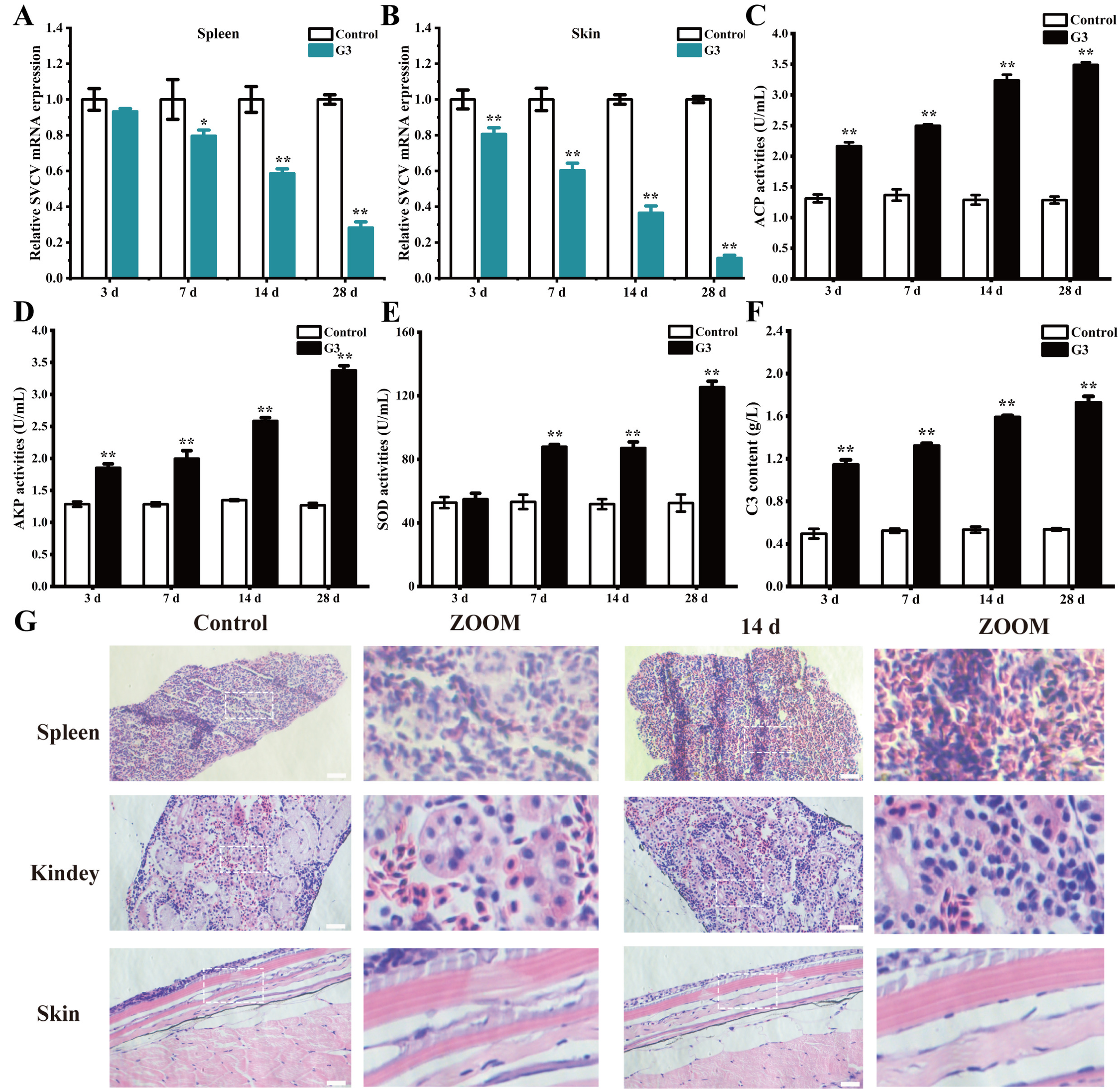

Supplement: S3 Fig — (A) The viral load in the spleen of vaccinated fish was quantified at different times post-vaccination using qPCR. (B) The viral load in the skin of vaccinated fish was quantified at different times post-vaccination using qPCR. (C) The ACP activities in the skin of vaccinated fish were measured at different times post-vaccination using ELISA. The ACP activities were significantly higher in G3 groups at 3 dpv (2.16 ± 0.06) and 7 dpv (2.5 ± 0.02) compared to the control group. (D) The AKP activities in the skin of vaccinated fish were measured at different times post-vaccination using ELISA. The AKP activities were significantly higher in G3 groups at 3 dpv (1.86 ± 0.06) and 7 dpv (2.0 ± 0.13) compared to the control group. (E) The SOD activities in the skin of vaccinated fish were measured at different times post-vaccination using ELISA. The SOD activities were significantly higher in G3 groups at 7 dpv (87.91 ± 1.43) compared to the control group. (F) The C3 content in the skin of vaccinated fish were measured at different times post-vaccination using ELISA. The C3 content were significantly higher in G3 groups at 3 dpv (1.15 ± 0.04) and 7 dpv (1.32 ± 0.02) compared to the control group. (G) Histological examination was performed using H&E staining of skin from vaccinated fish at 14 dpv. Scale bars, 50 μm. The P value for each study was calculated using Student’s t tests and one-way ANOVA. Statistical significance is indicated as follows: *P < 0.05, **P < 0.01. The data presented are representative of three independent experiments, with means ± standard error of the mean (SEM). (TIF) [file ppat.1012744.s003.tif]

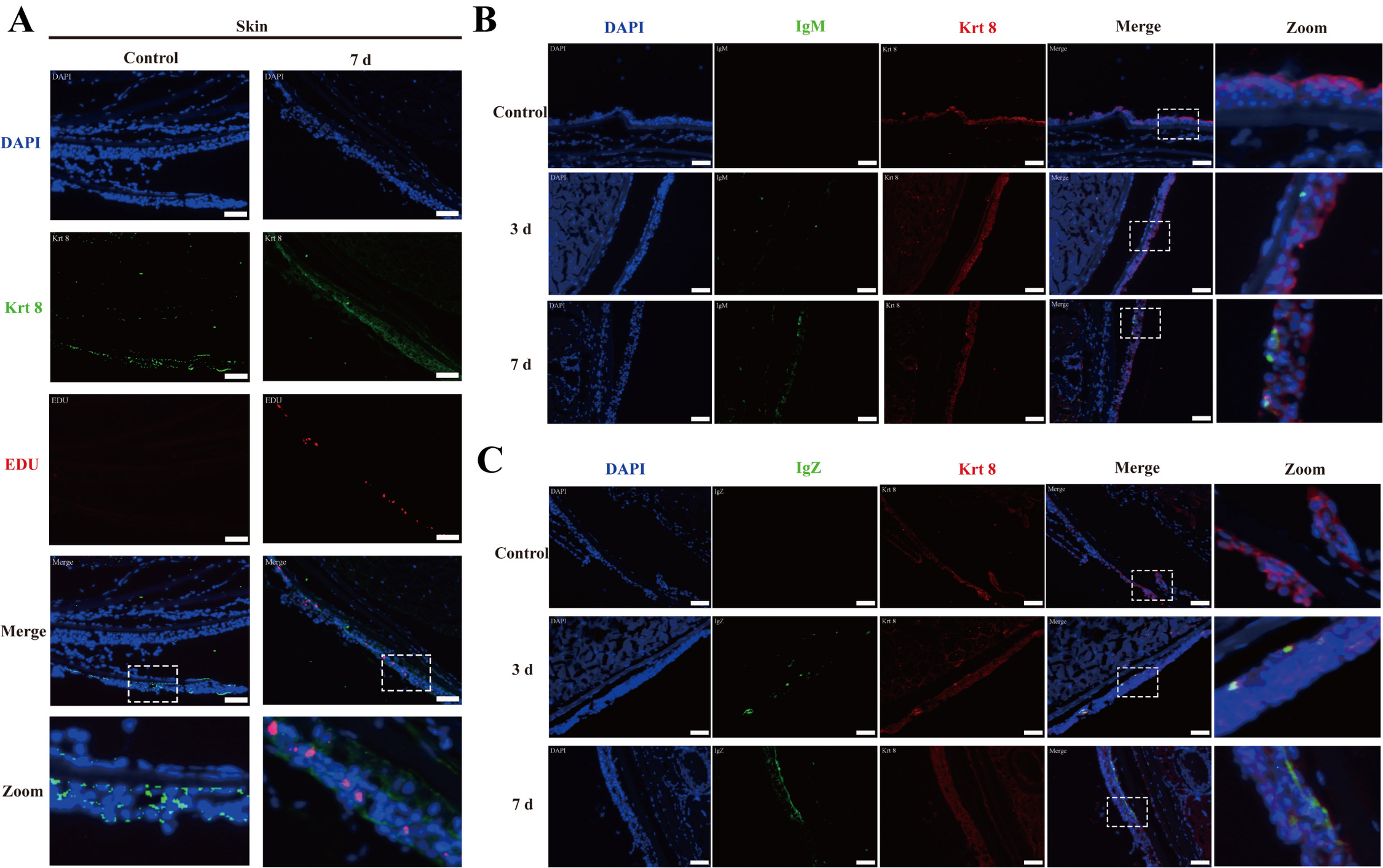

Supplement: S4 Fig — (A) Immunofluorescence analysis was performed to assess cell proliferation through EdU incorporation in the skin of vaccinated fish. EdU (red) was used to detect the cell proliferation in the skin tissue sections, Krt-8 (green) is used to label epidermal cells, nuclei were stained with DAPI (blue). Scale bars, 50 μm. (B) Representative immunofluorescence images for IgM (green) of skin from vaccinated fish on days 3 and 7 after immunization. Krt-8 (red) is used to label epidermal cells. Nuclei were stained with DAPI (blue). Scale bars, 50 μm. (C) Representative immunofluorescence images for IgZ (green) of skin from vaccinated fish on days 3 and 7 after immunization. Krt-8 (red) is used to label epidermal cells. Nuclei were stained with DAPI (blue). Scale bars, 50 μm. (TIF) [file ppat.1012744.s004.tif]

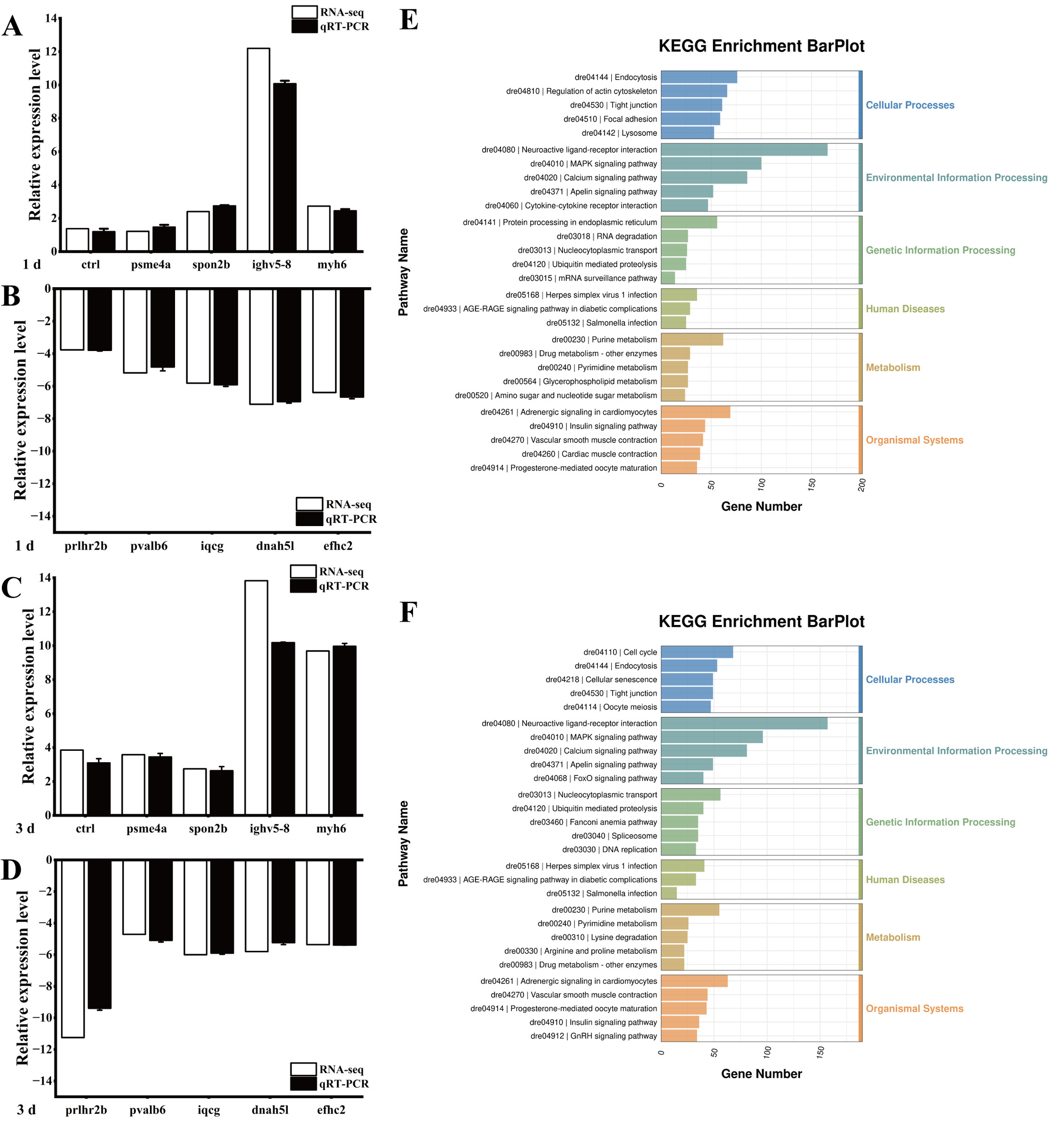

Supplement: S5 Fig — (A) Validation of transcriptomic data of upregulated genes at 1 dpv by qPCR. (B) Validation of transcriptomic data of downregulated genes at 1 dpv by qPCR. (C) Validation of transcriptomic data of upregulated genes at 3 dpv by qPCR. (D) Validation of transcriptomic data of downregulated genes at 3 dpv by qPCR. (E) The DEGs enrichment KEGG pathway analysis at 1 dpv compared with control group. (F) The DEGs enrichment KEGG pathway analysis at 3 dpv compared with control group. Data of qPCR are representative of three different independent experiment and shown as means ± SEM. (TIF) [file ppat.1012744.s005.tif]

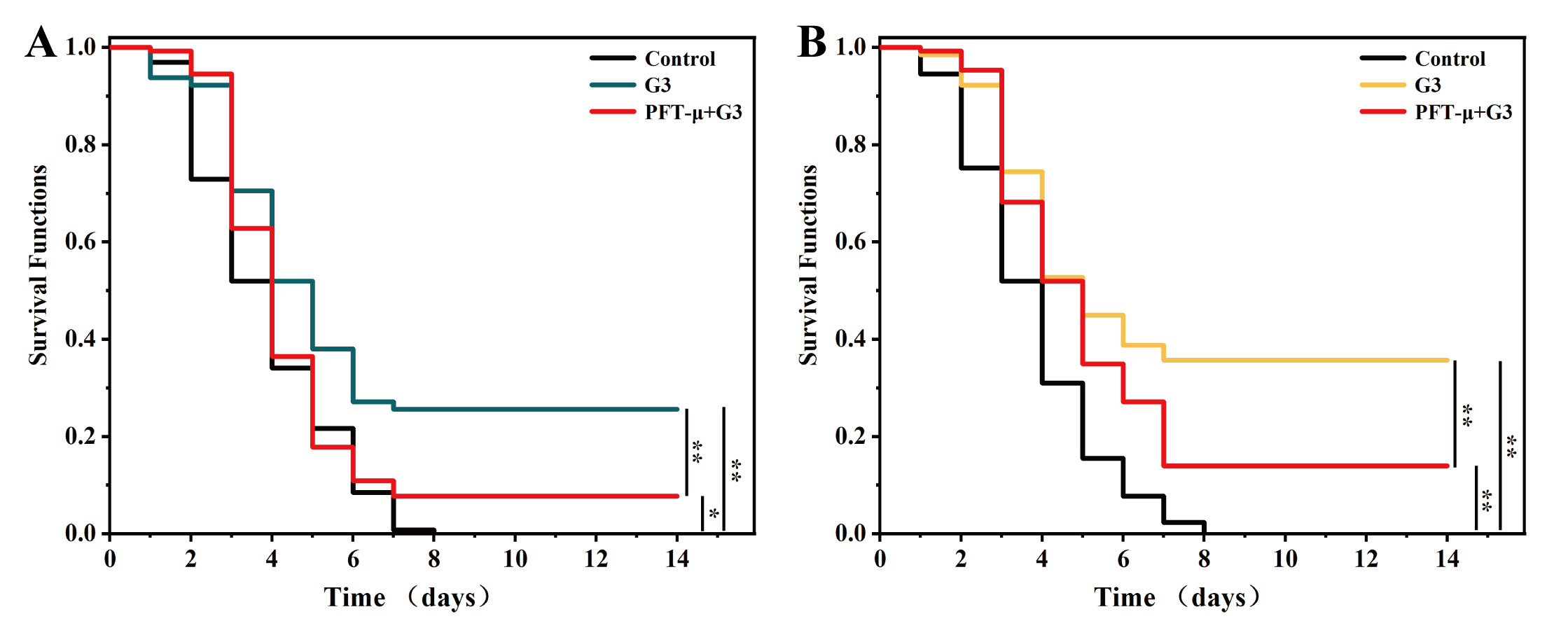

Supplement: S6 Fig — (A) After 3 days post-vaccination, the survival curve of vaccinated fish challenged with SVCV. (B) After 7 days post-vaccination, the survival curve of vaccinated fish challenged with SVCV. Survival analysis for each study was calculated using Kaplan-Meier method. Combine three replicates (43 fish per replicate) into one group for analysis. Log-rank test was performed for analyzing statistical significance, *P < 0.05, **P < 0.01. (TIF) [file ppat.1012744.s006.tif]
