# Supplementary material for: Skin as outermost immune organ of vertebrates that elicits robust early immune responses after immunization with glycoprotein of spring viraemia of carp virus
Source: PLoS Pathog. 2024 Dec 9;20(12):e1012744. doi: 10.1371/journal.ppat.1012744 (PMC11627376; doi:10.1371/journal.ppat.1012744)
Supplement: S1 Table — (DOCX) [file ppat.1012744.s007.docx]

**S1 Table** Amino acid sequences of G3 (glycoprotein, G^251-381^).

| Name of protein | Amino Acid Sequences |
| --- | --- |
| G3 (G^251-381^) | MGDWVEKTAETLTNIYANIPECADGTLVSGHRPGLDLIDTVFNLENVVEYTLCEGTKRKINNQEKLTSVDLSYLAPRIGGFGSVFRVRNGTLERGSTTYIKIEVEGPIVDSLNGTDPRTNASRVFWDDWEL |
