# Supplementary material for: Skin as outermost immune organ of vertebrates that elicits robust early immune responses after immunization with glycoprotein of spring viraemia of carp virus
Source: PLoS Pathog. 2024 Dec 9;20(12):e1012744. doi: 10.1371/journal.ppat.1012744 (PMC11627376; doi:10.1371/journal.ppat.1012744)
Supplement: S2 Table — (DOCX) [file ppat.1012744.s008.docx]

**S2 Table** Primers used for shRNA plasmid expressing HSP70-specific siRNAs.

| ShRNA | Primer sequences (from 5’ to 3’) | |
| --- | --- | --- |
| Si-HSP70-1 | Forward | CCGGTGGCAGATAAAGAGGAGTATTCTCGAGAATACTCCTCTTTATCTGCCATTTTTG |
|  | Reverse | AATTCAAAAATGGCAGATAAAGAGGAGTATTCTCGAGAATACTCCTCTTTATCTGCCA |
| Si-HSP70-2 | Forward | CCGGGGTGACCAACGCAGTTATCTTCTCGAGAAGATAACTGCGTTGGTCACCTTTTTG |
|  | Reverse | AATTCAAAAAGGTGACCAACGCAGTTATCTTCTCGAGAAGATAACTGCGTTGGTCACC |
| Si-HSP70-3 | Forward | CCGGACAACCAGCTGGCAGATAATTCTCGAGAATTATCTGCCAGCTGGTTGTTTTTTG |
|  | Reverse | AATTCAAAAAACAACCAGCTGGCAGATAATTCTCGAGAATTATCTGCCAGCTGGTTGT |
| Si-NC | Forward | CCGGCAACAAGATGAAGAGCACCAACTCGAGTTGGTGCTCTTCATCTTGTTGTTTTTG |
|  | Reverse | AATTCAAAAACAACAAGATGAAGAGCACCAACTCGAGTTGGTGCTCTTCATCTTGTTG |
