# Supplementary material for: Skin as outermost immune organ of vertebrates that elicits robust early immune responses after immunization with glycoprotein of spring viraemia of carp virus
Source: PLoS Pathog. 2024 Dec 9;20(12):e1012744. doi: 10.1371/journal.ppat.1012744 (PMC11627376; doi:10.1371/journal.ppat.1012744)
Supplement: S3 Table — (DOCX) [file ppat.1012744.s009.docx]

**S3 Table** Primers used for the analysis of mRNA expression by qPCR.

| Genes | Accession no. | | Primer sequences (from 5’ to 3’) | Product size (bp) |
| --- | --- | --- | --- | --- |
| *β-actin* | NM_131031.2 | Forward | AGGTCATCACCATCGGCAAT | 130 |
|  |  | Reverse | GATGTCCACGTCGCACTTCA | |
| *znf687a* | NM_001353864.1 | Forward | CCTGGCAGAAAAATGGACAAG | 163 |
|  |  | Reverse | CCAACAACACCAATGACAATC | |
| *Sncga* | NM_001017567.2 | Forward | GACCAGGCAAATCTCAT | 133 |
|  |  | Reverse | GCTCCTCGTGCTTCA | |
| *ephb4a* | NM_131414.1 | Forward | TCCACTCACGCCCTCA | 224 |
|  |  | Reverse | TCCCTGGTGCTTTGTTT | |
| *ube2l3a* | NM_001002072.2 | Forward | GAGAAGGGTCAGGTGT | 112 |
|  |  | Reverse | CTGGTTGCGGGTC | |
| *impa1* | NM_001002745.1 | Forward | GCAGAACGACCTGAAGA | 242 |
|  |  | Reverse | ATACGGCAACAAATGG | |
| *dnai2a* | NM_001110465.1 | Forward | GAGTGAGCCCACAAAGC | 261 |
|  |  | Reverse | TCAGCCACAGTCAAGAAGTA | |
| *eif5a* | NM_213185.1 | Forward | ACGGGTTTGTGGTGCT | 153 |
|  |  | Reverse | TGTTGTGGGTGGAGGG |  |
| *CR855996* | XM_686811.7 | Forward | TGTCTATCGGGAACG | 104 |
|  |  | Reverse | GGTGAGAAACGCTGA |  |
| *ulk4* | XM_009292054.3 | Forward | AACAAATGCGACAGGC | 262 |
|  |  | Reverse | GCAGGAACGACCCAC |  |
| *cyp2k19* | NM_001079704.2 | Forward | AAAACCGTCAAAGAAGC | 169 |
|  |  | Reverse | CCATCCCAAAGTCCC |  |
| *HSP70* | NM_001113589.1 | Forward | TCACGGACACCGAAAG | 117 |
|  |  | Reverse | CCACTGGGTCATCAAAGC |  |
| *Hsc70* | NM_200614.1 | Forward | ATGAACCCTTCTAACAC | 266 |
|  |  | Reverse | TAAGCAGGCACCGTAAT |  |
| *Hsp70l* | NM_001113589.1 | Forward | TGATGACCCTGTAGTGC | 229 |
|  |  | Reverse | CTGGGAGTCGTTGAAATA |  |
| *Hspa8b* | NM_001200012.1 | Forward | GAGGCTGTTGCGTATGG | 194 |
|  |  | Reverse | TAGGTGGTGAAGGTCTGAGTT |  |
| *Hspa8* | NM_001110403.1 | Forward | TTCAAACGCAAGCACAA | 204 |
|  |  | Reverse | GCCACGGAAGAGGTCAG |  |
| *Hspa1b* | NM_001100062.1 | Forward | GTGGCTCCGTTGTCCCT | 127 |
|  |  | Reverse | CTCCCGGCTGGTTATCG |  |
| *Hsp90* | NM_198210.2 | Forward | TGGCGTTCGCATCTGTA | 294 |
|  |  | Reverse | AGGCATCGGAAGCATTA |  |
| *Hspa9* | NM_201326.2 | Forward | CTTTAGCCTATGGACTG | 188 |
|  |  | Reverse | CTCTTTGACTATGTGCC |  |
| *Hspa14* | NM_001045076.1 | Forward | AGTTTACCGTGTTCTCG | 119 |
|  |  | Reverse | CACTCACATCCTGCTTA |  |
| *Hspa5* | NM_213058.1 | Forward | CCGTTCCTGCTTATTTC | 243 |
|  |  | Reverse | GAGTGTCTCCGTTTGTG |  |
| *Hspa13* | NM_001089479.1 | Forward | TCCTGGTTGGAGGGTCT | 131 |
|  |  | Reverse | AATGCCTGCCTGAATGG |  |
| *ERK* | NM_182888.2 | Forward | ACCTGAAGCCCTCAAACC | 203 |
|  |  | Reverse | GCATCCCACAGACCAAAT |  |
| *JNK* | NM_001037701.2 | Forward | GCAGACTCGGACCACAAC | 250 |
|  |  | Reverse | CCTCCCATTCCAGCACTT |  |
| *P38* | XM_001337797.8 | Forward | TCGTCAAGTGCCAGAAGC | 168 |
|  |  | Reverse | CCAACCCAAAGTCCAAAA |  |
| *CD36* | NM_001002363.1 | Forward | CTGGCAAGGTGAAACAT | 234 |
|  |  | Reverse | TTGGAAACTGGAGAAGC |  |
| *TAP1* | XM_002665007.3 | Forward | TCACAGCACTCGTGGGTC | 218 |
|  |  | Reverse | GTTTTCTTTGCCGTATTTG |  |
| *IL-1β* | NM_212844.2 | Forward | CCCCAATCCACAGAGTTT | 76 |
|  |  | Reverse | TTCACTTCACGCTCTTGG |  |
| *IL-6* | NM_001261449.1 | Forward | ACGGAAAGATGTCTAACGC | 197 |
|  |  | Reverse | GATAGGGAAGTGCTGGATG |  |
| *IL-12* | XM_017352586.2 | Forward | CCGAAGGAAAGAGTATCACC | 301 |
|  |  | Reverse | CTCAGTTGGGAGCAGTCA |  |
| *MyD88* | DQ100359.1 | Forward | TGGAGATCAAAAACTT | 164 |
|  |  | Reverse | TGCAGTCGTCATCTAAAATTTCT |  |
| *NF-κβ* | NM_001001839.2 | Forward | GTGGTTCGGCTGATGTTC | 394 |
|  |  | Reverse | GTTCGCTCGTCTCGTTGT |  |
| *TGF-β* | NM_182873.1 | Forward | ATCTGGGTTGGAAGTGG | 173 |
|  |  | Reverse | GTCAAGGATTGCGGGTA |  |
| *TNF-α* | NM_212859.2 | Forward | CATTTTGGCTGTGGGCCTTTGTG | 263 |
|  |  | Reverse | TCATCGGGAATGATAATCTC |  |
| *TLR2* | AY388399 | Forward | AGATGTCTCCCACCCTG | 104 |
|  |  | Reverse | TCTTAGTGCCACCTTCCT |  |
| *TLR4* | NM_001131051 | Forward | ATCTCAGACAGCTGGATATTC | 204 |
|  |  | Reverse | CTTTCCAAAACATAGTAAGATAC |  |
| *TRAF2* | XM_005171946.4 | Forward | TTTTCCTGATAATGCTGCTA | 100 |
|  |  | Reverse | TGCCTCGTATTCTTTGAT |  |
| *TRAF3* | NM_001003513.1 | Forward | AAGAAACCGTCTGTCCTG | 138 |
|  |  | Reverse | ATCCGTAGCGAATGAAG |  |
| *TRAF6* | NM_001044752.1 | Forward | GTGCAGGTTTGCCACTG | 206 |
|  |  | Reverse | ATGAGCTCCATTTCGCAATATTC |  |
| *SVCV-N* | KJ513477.1 | Forward | AACAGCGCGTCTTACATGC | 208 |
|  |  | Reverse | CTAAGGCGTAAGCCATCAGC | |
